# Genetic assessment of age-associated Alzheimer disease risk: Development and validation of a polygenic hazard score

**DOI:** 10.1371/journal.pmed.1002258

**Published:** 2017-03-21

**Authors:** Rahul S. Desikan, Chun Chieh Fan, Yunpeng Wang, Andrew J. Schork, Howard J. Cabral, L. Adrienne Cupples, Wesley K. Thompson, Lilah Besser, Walter A. Kukull, Dominic Holland, Chi-Hua Chen, James B. Brewer, David S. Karow, Karolina Kauppi, Aree Witoelar, Celeste M. Karch, Luke W. Bonham, Jennifer S. Yokoyama, Howard J. Rosen, Bruce L. Miller, William P. Dillon, David M. Wilson, Christopher P. Hess, Margaret Pericak-Vance, Jonathan L. Haines, Lindsay A. Farrer, Richard Mayeux, John Hardy, Alison M. Goate, Bradley T. Hyman, Gerard D. Schellenberg, Linda K. McEvoy, Ole A. Andreassen, Anders M. Dale

**Affiliations:** 1 Neuroradiology Section, Department of Radiology and Biomedical Imaging, University of California, San Francisco, California, United States of America; 2 Department of Cognitive Science, University of California, San Diego, La Jolla, California, United States of America; 3 Department of Neurosciences, University of California, San Diego, La Jolla, California, United States of America; 4 Norwegian Centre for Mental Disorders Research (NORMENT), Institute of Clinical Medicine, University of Oslo, Oslo, Norway; 5 Division of Mental Health and Addiction, Oslo University Hospital, Oslo, Norway; 6 Department of Biostatistics, Boston University School of Public Health, Boston, Massachusetts, United States of America; 7 Institute for Biological Psychiatry, Sankt Hans Psychiatric Hospital, Roskilde, Denmark; 8 National Alzheimer’s Coordinating Center, Department of Epidemiology, University of Washington, Seattle, Washington, United States of America; 9 Department of Radiology, University of California, San Diego, La Jolla, California, United States of America; 10 Shiley-Marcos Alzheimer’s Disease Research Center, University of California, San Diego, La Jolla, California, United States of America; 11 Department of Psychiatry, Washington University, St. Louis, Missouri, United States of America; 12 Department of Neurology, University of California, San Francisco, California, United States of America; 13 John P. Hussman Institute for Human Genomics, University of Miami, Miami, Florida, United States of America; 14 Department of Epidemiology and Biostatistics, Case Western University, Cleveland, Ohio, United States of America; 15 Institute for Computational Biology, Case Western University, Cleveland, Ohio, United States of America; 16 Department of Medicine (Biomedical Genetics), Boston University School of Medicine, Boston, Massachusetts, United States of America; 17 Department of Neurology, Boston University School of Medicine, Boston, Massachusetts, United States of America; 18 Department of Ophthalmology, Boston University School of Medicine, Boston, Massachusetts, United States of America; 19 Department of Biostatistics, Boston University School of Public Health, Boston, Massachusetts, United States of America; 20 Department of Epidemiology, Boston University School of Public Health, Boston, Massachusetts, United States of America; 21 Department of Neurology, Columbia University, New York, New York, United States of America; 22 Taub Institute on Alzheimer’s Disease and the Aging Brain, Columbia University, New York, New York, United States of America; 23 Gertrude H. Sergievsky Center, Columbia University, New York, New York, United States of America; 24 Department of Molecular Neuroscience, UCL Institute of Neurology, University College London, London, United Kingdom; 25 Department of Neuroscience, Icahn School of Medicine at Mount Sinai, New York, New York, United States of America; 26 Department of Genetics and Genomic Sciences, Icahn School of Medicine at Mount Sinai, New York, New York, United States of America; 27 Department of Neurology, Massachusetts General Hospital, Boston, Massachusetts, United States of America; 28 Department of Pathology and Laboratory Medicine, University of Pennsylvania Perelman School of Medicine, Philadelphia, Pennsylvania, United States of America; University of Cambridge, UNITED KINGDOM

## Abstract

**Background:**

Identifying individuals at risk for developing Alzheimer disease (AD) is of utmost importance. Although genetic studies have identified AD-associated SNPs in *APOE* and other genes, genetic information has not been integrated into an epidemiological framework for risk prediction.

**Methods and findings:**

Using genotype data from 17,008 AD cases and 37,154 controls from the International Genomics of Alzheimer’s Project (IGAP Stage 1), we identified AD-associated SNPs (at *p <* 10^−5^). We then integrated these AD-associated SNPs into a Cox proportional hazard model using genotype data from a subset of 6,409 AD patients and 9,386 older controls from Phase 1 of the Alzheimer’s Disease Genetics Consortium (ADGC), providing a polygenic hazard score (PHS) for each participant. By combining population-based incidence rates and the genotype-derived PHS for each individual, we derived estimates of instantaneous risk for developing AD, based on genotype and age, and tested replication in multiple independent cohorts (ADGC Phase 2, National Institute on Aging Alzheimer’s Disease Center [NIA ADC], and Alzheimer’s Disease Neuroimaging Initiative [ADNI], total *n* = 20,680). Within the ADGC Phase 1 cohort, individuals in the highest PHS quartile developed AD at a considerably lower age and had the highest yearly AD incidence rate. Among *APOE* ε3/3 individuals, the PHS modified expected age of AD onset by more than 10 y between the lowest and highest deciles (hazard ratio 3.34, 95% CI 2.62–4.24, *p* = 1.0 × 10^−22^). In independent cohorts, the PHS strongly predicted empirical age of AD onset (ADGC Phase 2, *r* = 0.90, *p* = 1.1 × 10^−26^) and longitudinal progression from normal aging to AD (NIA ADC, Cochran–Armitage trend test, *p* = 1.5 × 10^−10^), and was associated with neuropathology (NIA ADC, Braak stage of neurofibrillary tangles, *p* = 3.9 × 10^−6^, and Consortium to Establish a Registry for Alzheimer’s Disease score for neuritic plaques, *p* = 6.8 × 10^−6^) and in vivo markers of AD neurodegeneration (ADNI, volume loss within the entorhinal cortex, *p* = 6.3 × 10^−6^, and hippocampus, *p* = 7.9 × 10^−5^). Additional prospective validation of these results in non-US, non-white, and prospective community-based cohorts is necessary before clinical use.

**Conclusions:**

We have developed a PHS for quantifying individual differences in age-specific genetic risk for AD. Within the cohorts studied here, polygenic architecture plays an important role in modifying AD risk beyond *APOE*. With thorough validation, quantification of inherited genetic variation may prove useful for stratifying AD risk and as an enrichment strategy in therapeutic trials.

## Abstract 2

Rahul Desikan and colleagues use genetic and epidemiological data from several large cohorts to derive a score for predicting the age-specific risk for developing Alzheimer's disease.

## Author summary

**Why was this study done?:**

- Across the United States, late-onset Alzheimer’s disease (AD) is the most common form of dementia.
- There is a strong need for in vivo markers for AD risk stratification and cohort enrichment in therapeutic trials.
- Although numerous studies have identified several genetic risk factors, including the ε4 allele of *apolipoprotein E* (*APOE*), genetic variants have not been integrated with genetic epidemiology for quantifying age of AD onset.

**What did the researchers do and find?:**

- Using genotype data from over 70,000 AD patients and normal elderly controls, we evaluated the feasibility of combining AD-associated SNPs and *APOE* status into a continuous measure—a polygenic hazard score (PHS)—for predicting the age-specific risk for developing AD.
- Using a survival model framework, we integrated single nucleotide polymorphisms associated with increased risk for AD into a PHS for each participant. By combining population-based incidence rates and the genotype-derived PHS for each individual, we derived estimates of instantaneous risk for developing AD, based on genotype and age, and tested replication in two independent cohorts.
- Individuals in the highest PHS quartile developed AD at a considerably lower age and had the highest yearly AD incidence rate.
- In independent cohorts, we found that the PHS strongly predicted empirical age of AD onset and longitudinal progression from normal aging to AD, and associated strongly with neuropathology and in vivo markers of AD neurodegeneration.
- Additional prospective validation of these results on non-US, non-white, and prospective community-based cohorts is necessary before clinical use.

**What do these findings mean?:**

- Genetic variants can be integrated within an epidemiology framework to derive a polygenic score that can quantify individual differences in age-specific genetic risk for AD, beyond *APOE*.
- Quantification of inherited genetic variation may prove useful for AD risk stratification and for therapeutic trials.

## Introduction

Late-onset Alzheimer disease (AD), the most common form of dementia, places a large emotional and economic burden on patients and society. With increasing health care expenditures among cognitively impaired elderly individuals [1], identifying individuals at risk for developing AD is of utmost importance for potential preventative and therapeutic strategies. Inheritance of the ε4 allele of *apolipoprotein E* (*APOE*) on Chromosome 19q13 is the most significant risk factor for developing late-onset AD [2]. *APOE* ε4 has a dose-dependent effect on age of onset, increases AD risk 3-fold in heterozygotes and 15-fold in homozygotes, and is implicated in 20%–25% of AD cases [3].

In addition to the single nucleotide polymorphism (SNP) in *APOE*, recent genome-wide association studies (GWASs) have identified numerous AD-associated SNPs, most of which have a small effect on disease risk [4,5]. Although no single polymorphism may be informative clinically, a combination of *APOE* and non-*APOE* SNPs may help identify older individuals at increased risk for AD. Despite their detection of novel AD-associated genes, GWAS findings have not yet been incorporated into a genetic epidemiology framework for individualized risk prediction.

Building on a prior approach evaluating GWAS-detected genetic variants for disease prediction [6] and using a survival analysis framework, we tested the feasibility of combining AD-associated SNPs and *APOE* status into a continuous-measure polygenic hazard score (PHS) for predicting the age-specific risk for developing AD. We assessed replication of the PHS using several independent cohorts.

## Methods

### Participant samples

#### International genomics of Alzheimer’s project

To select AD-associated SNPs, we evaluated publicly available AD GWAS summary statistic data (*p-*values and odds ratios) from the International Genomics of Alzheimer’s Project (IGAP) (Stage 1; for additional details see S1 Appendix and [4]). For selecting AD-associated SNPs, we used IGAP Stage 1 data, from 17,008 AD cases and 37,154 controls drawn from four different consortia across North America and Europe (including the United States of America, England, France, Holland, and Iceland) with genotyped or imputed data at 7,055,881 SNPs (for a description of the AD cases and controls within the IGAP Stage 1 sub-studies, please see Table 1 and [4]).

**Table 1:** Demographic data for AD patients and older controls.

Characteristic	IGAP		ADGC Phase 1		ADGC Phase 2	
	AD patients	Older controls	AD patients	Older controls	AD patients	Older controls
Total *N*	17,008	37,154	6,409	9,386	6,984	10,972
Mean age (SD) of onset (cases) or assessment (controls)	74.7 (8.0)	68.6 (8.5)	74.7 (7.7)	76.4 (8.1)	73.6 (7.3)	75.7 (8.6)
Percent female	63.0%	57.0%	61.0%	59.0%	57.6%	60.7%
Percent *APOE* ε4 carriers	59.0%	25.4%	51.6%	26.7%	56.0%	28.4%

ADGC, Alzheimer’s Disease Genetics Consortium; IGAP, International Genomics of Alzheimer’s Project; SD, standard deviation.

#### Alzheimer’s disease genetics consortium

To develop the survival model for the PHS, we first evaluated age of onset and raw genotype data from 6,409 patients with clinically diagnosed AD and 9,386 cognitively normal older individuals provided by the Alzheimer’s Disease Genetics Consortium (ADGC) (Phase 1, a subset of the IGAP dataset), excluding individuals from the National Institute of Aging Alzheimer’s Disease Center (NIA ADC) and Alzheimer’s Disease Neuroimaging Initiative (ADNI) samples. To evaluate replication of the PHS, we used an independent sample of 6,984 AD patients and 10,972 cognitively normal older individuals from the ADGC Phase 2 cohort (Table 1). The genotype and phenotype data within the ADGC datasets has been described in detail elsewhere [7,8]. Briefly, the ADGC Phase 1 and 2 datasets (enrollment from 1984 to 2012) consist of case–control, prospective, and family-based sub-studies of white participants with AD occurrence after age 60 y derived from the general community and Alzheimer’s Disease Centers across the US. Participants with autosomal dominant (*APP*, *PSEN1*, and *PSEN2*) mutations were excluded. All participants were genotyped using commercially available high-density SNP microarrays from Illumina or Affymetrix. Clinical diagnosis of AD within the ADGC sub-studies was established using NINCDS-ADRDA criteria for definite, probable, and possible AD [9]. For most participants, age of AD onset was obtained from medical records and defined as the age when AD symptoms manifested, as reported by the participant or an informant. For participants lacking age of onset, age at ascertainment was used. Patients with an age at onset or age at death less than 60 y and individuals of non-European ancestry were excluded from the analyses. All ADGC Phase 1 and 2 control participants were defined within individual sub-studies as cognitively normal older adults at time of clinical assessment. The institutional review boards of all participating institutions approved the procedures for all ADGC sub-studies. Written informed consent was obtained from all participants or surrogates. For additional details regarding the ADGC datasets, please see [7,8].

#### National institute of aging Alzheimer’s disease centers

To assess longitudinal prediction, we evaluated an ADGC-independent sample of 2,724 cognitively normal elderly individuals. Briefly, all participants were US based, evaluated at National Institute of Aging–funded Alzheimer’s Disease Centers (data collection coordinated by the National Alzheimer’s Coordinating Center [NACC]) and clinically followed for at least two years (enrollment from 1984 to 2012, evaluation years were 2005 to 2016) [10]. Here, we focused on older individuals defined at baseline as having an overall Clinical Dementia Rating score of 0.0. To assess the relationship between polygenic risk and neuropathology, we assessed 2,960 participants from the NIA ADC samples with genotype and neuropathological evaluations. For the neuropathological variables, we examined the Braak stage for neurofibrillary tangles (NFTs) (0, none; I–II, entorhinal; III–IV, limbic; and V–VI, isocortical) [11] and the Consortium to Establish a Registry for Alzheimer’s Disease (CERAD) score for neuritic plaques (none/sparse, moderate, or frequent) [12]. Finally, as an additional independent replication sample, we evaluated all NIA ADC AD cases with genetic data who were classified at autopsy as having a high level of AD neuropathological change (*n* = 361), based on the revised National Institute of Aging–Alzheimer’s Association AD neuropathology criteria [13]. The institutional review boards of all participating institutions approved the procedures for all NIA ADC sub-studies. Written informed consent was obtained from all participants or surrogates.

#### Alzheimer’s disease neuroimaging initiative

To assess the relationship between polygenic risk and in vivo biomarkers, we evaluated an ADGC-independent sample of 692 older controls and participants with mild cognitive impairment or AD from the ADNI (see S1 Appendix). Briefly, the ADNI is a multicenter, multisite longitudinal study assessing clinical, imaging, genetic, and biospecimen biomarkers from US-based participants through the process of normal aging to early mild cognitive impairment, to late mild cognitive impairment, to dementia or AD (see S1 Appendix). Here, we focused specifically on participants from ADNI 1 with cognitive, imaging, and cerebrospinal fluid (CSF) assessments from 2003 to 2010. In a subset of ADNI 1 participants with available genotype data, we evaluated baseline CSF level of Aβ_1–42_ and total tau, as well as longitudinal Clinical Dementia Rating Sum of Boxes (CDR-SB) scores. In ADNI 1 participants with available genotype and quality-assured baseline and follow-up MRI scans, we also assessed longitudinal subregional change in medial temporal lobe volume (atrophy) on 2,471 serial T_1_-weighted MRI scans (for additional details see S1 Appendix).

### Statistical analysis

We followed three steps to derive the PHS for predicting age of AD onset: (1) we defined the set of associated SNPs, (2) we estimated hazard ratios for polygenic profiles, and (3) we calculated individualized absolute hazards (see S1 Appendix for a detailed description of these steps).

Using the IGAP Stage 1 sample, we first identified a list of SNPs associated with increased risk for AD, using a significance threshold of *p <* 10^−5^. Next, we evaluated all IGAP-detected AD-associated SNPs within the ADGC Phase 1 case–control dataset. Using a stepwise procedure in survival analysis, we delineated the “final” list of SNPs for constructing the PHS [14,15]. Specifically, using Cox proportional hazard models, we identified the top AD-associated SNPs within the ADGC Phase 1 cohort (excluding NIA ADC and ADNI samples), while controlling for the effects of gender, *APOE* variants, and the top five genetic principal components (to control for the effects of population stratification). We utilized age of AD onset and age of last clinical visit to estimate age-specific risks [16] and derived a PHS for each participant. In each step of the stepwise procedure, the algorithm selected the one SNP from the pool that most improved model prediction (i.e., minimizing the Martingale residuals); additional SNP inclusion that did not further minimize the residuals resulted in halting of the SNP selection process. To prevent overfitting in this training step, we used 1,000× bootstrapping for model averaging and estimating the hazard ratios for each selected SNP. We assessed the proportional hazard assumption in the final model using graphical comparisons.

To assess for replication, we first examined whether the predicted PHSs derived from the ADGC Phase 1 cohort could stratify individuals into different risk strata within the ADGC Phase 2 cohort. We next evaluated the relationship between predicted age of AD onset and the empirical (actual) age of AD onset using cases from ADGC Phase 2. We binned risk strata into percentile bins and calculated the mean of actual age of AD onset in that percentile as the empirical age of AD onset. In a similar fashion, we additionally tested replication within the NIA ADC subset classified at autopsy as having a high level of AD neuropathological change [13].

Because case–control samples cannot provide the proper baseline hazard [17], we used previously reported annualized incidence rates by age estimated from the general US population [18]. For each participant, by combining the overall population-derived incidence rates [18] and the genotype-derived PHS, we calculated the individual’s “instantaneous risk” for developing AD, based on their genotype and age (for additional details see S1 Appendix). To independently assess the predicted instantaneous risk, we evaluated longitudinal follow-up data from 2,724 cognitively normal older individuals from the NIA ADC with at least 2 y of clinical follow-up. We assessed the number of cognitively normal individuals progressing to AD as a function of the predicted PHS risk strata and examined whether the predicted PHS-derived incidence rate reflected the empirical progression rate using a Cochran–Armitage trend test.

We examined the association between our PHS and established in vivo and pathological markers of AD neurodegeneration. Using linear models, we assessed whether the PHS associated with Braak stage for NFTs and CERAD score for neuritic plaques, as well as CSF Aβ_1–42_ and CSF total tau. Using linear mixed effects models, we also investigated whether the PHS was associated with longitudinal CDR-SB score and volume loss within the entorhinal cortex and hippocampus. In all analyses, we co-varied for the effects of age and sex.

## Results

### Polygenic hazard score: Model development, relationship to *APOE*, and independent replication

**Fig 1:**
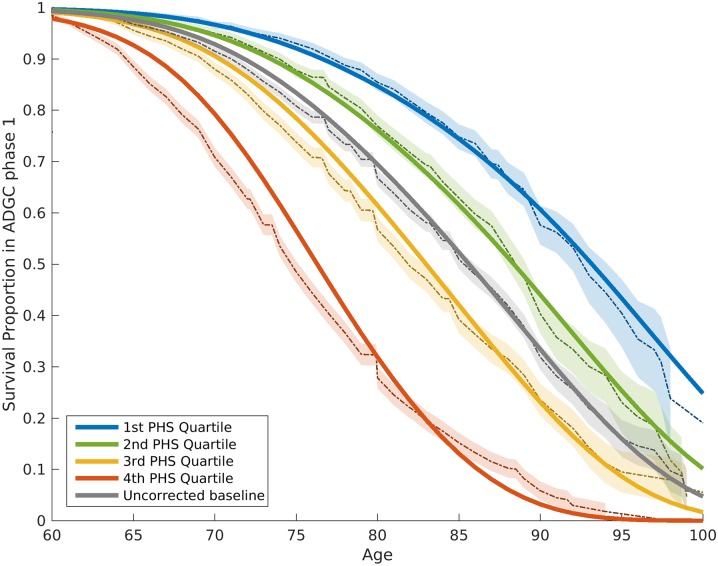
Kaplan–Meier estimates and Cox proportional hazard model fits from the ADGC Phase 1 case–control dataset, excluding NIA ADC and ADNI samples. The proportional hazard assumptions were checked based on graphical comparisons between Kaplan–Meier estimations (dashed lines) and Cox proportional hazard models (solid lines). The 95% confidence intervals of Kaplan–Meier estimations are also demonstrated (shaded with corresponding colors). The baseline hazard (gray line) in this model is based on the mean of ADGC data. ADGC, Alzheimer’s Disease Genetics Consortium; ADNI, Alzheimer’s Disease Neuroimaging Initiative; NIA ADC, National Institute on Aging Alzheimer’s Disease Center; PHS, polygenic hazard score.

From the IGAP cohort, we found 1,854 SNPs associated with increased risk for AD at *p <* 10^−5^. Of these, using the Cox stepwise regression framework, we identified 31 SNPs, in addition to two *APOE* variants, within the ADGC cohort for constructing the polygenic model (Table 2). Fig 1 illustrates the relative risk for developing AD using the ADGC Phase 1 case–control cohort. The graphical comparisons among Kaplan–Meier estimations and Cox proportional hazard models indicate that the proportional hazard assumption holds for the final model (Fig 1).

**Table 2:** The selected 31 SNPs, their closest genes, their log hazard ratio estimates, and their conditional *p-*values in the final joint model, after controlling for effects of gender and *APOE* variants.

SNP	Chromosome	Position	Gene	β (log HR)	Conditional *p-*value in −log_10_
ε2 allele	19		*APOE*	−0.47	>15.0
ε4 allele	19		*APOE*	1.03	>20.0
rs4266886	1	207685786	*CR1*	−0.09	2.7
rs61822977	1	207796065	*CR1*	−0.08	2.8
rs6733839	2	127892810	*BIN1*	−0.15	10.5
rs10202748	2	234003117	*INPP5D*	−0.06	2.1
rs115124923	6	32510482	*HLA-DRB5*	0.17	7.4
rs115675626	6	32669833	*HLA-DQB1*	−0.11	3.2
rs1109581	6	47678182	*GPR115*	−0.07	2.6
rs17265593	7	37619922	*BC043356*	−0.23	3.6
rs2597283	7	37690507	*BC043356*	0.28	4.7
rs1476679	7	100004446	*ZCWPW1*	0.11	4.9
rs78571833	7	143122924	*AL833583*	0.14	3.8
rs12679874	8	27230819	*PTK2B*	−0.09	4.2
rs2741342	8	27330096	*CHRNA2*	0.09	2.9
rs7831810	8	27430506	*CLU*	0.09	3.0
rs1532277	8	27466181	*CLU*	0.21	8.3
rs9331888	8	27468862	*CLU*	0.16	5.1
rs7920721	10	11720308	*CR595071*	−0.07	2.9
rs3740688	11	47380340	*SPI1*	0.07	2.8
rs7116190	11	59964992	*MS4A6A*	0.08	3.9
rs526904	11	85811364	*PICALM*	−0.20	2.3
rs543293	11	85820077	*PICALM*	0.30	4.2
rs11218343	11	121435587	*SORL1*	0.18	2.8
rs6572869	14	53353454	*FERMT2*	−0.11	3.0
rs12590273	14	92934120	*SLC24A4*	0.10	3.5
rs7145100	14	107160690	*abParts*	0.08	2.0
rs74615166	15	64725490	*TRIP4*	−0.23	3.1
rs2526378	17	56404349	*BZRAP1*	0.09	4.9
rs117481827	19	1021627	*C19orf6*	−0.09	2.5
rs7408475	19	1050130	*ABCA7*	0.18	4.3
rs3752246	19	1056492	*ABCA7*	−0.25	8.4
rs7274581	20	55018260	*CASS4*	0.10	2.1

**Fig 2:**
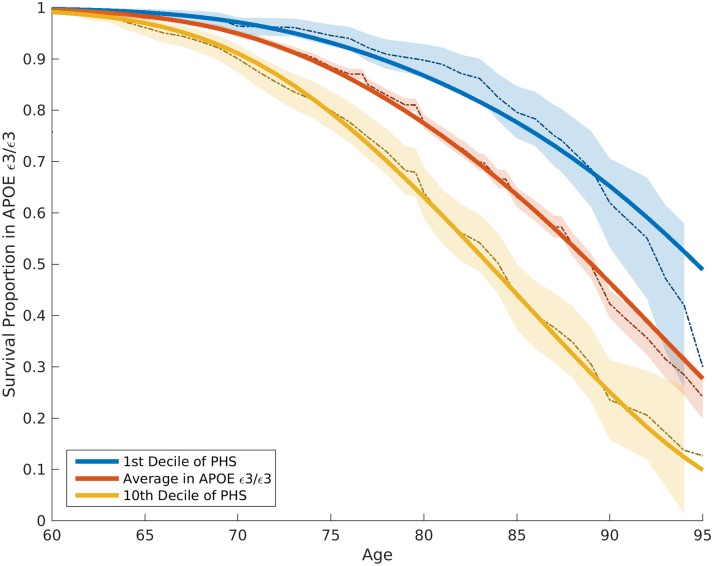
Kaplan–Meier estimates and Cox proportional hazard model fits among *APOE* ε3/3 individuals in the ADGC Phase 1 dataset, excluding NIA ADC and ADNI samples. The solid lines represent the Cox fit, whereas the dashed lines and shaded regions represent the Kaplan–Meier estimations with 95% confidence intervals. ADGC, Alzheimer’s Disease Genetics Consortium; ADNI, Alzheimer’s Disease Neuroimaging Initiative; NIA ADC, National Institute on Aging Alzheimer’s Disease Center; PHS, polygenic hazard score.

To quantify the additional prediction provided by polygenic information beyond *APOE*, we evaluated how the PHS modulates age of AD onset in *APOE* ε3/3 individuals. Among these individuals, we found that age of AD onset can vary by more than 10 y, depending on polygenic risk. For example, for an *APOE* ε3/3 individual in the tenth decile (top 10%) of the PHS, at 50% risk for meeting clinical criteria for AD diagnosis, the expected age of developing AD is approximately 84 y (Fig 2); however, for an *APOE* ε3/3 individual in the first decile (bottom 10%) of the PHS, the expected age of developing AD is approximately 95 y (Fig 2). The hazard ratio comparing the tenth decile to the first decile is 3.34 (95% CI 2.62–4.24, log rank test *p* = 1.0 × 10^−22^). Similarly, we also evaluated the relationship between the PHS and the different *APOE* alleles (ε2/3/4) (first figure in S1 Appendix). These findings show that, beyond *APOE*, the polygenic architecture plays an integral role in affecting AD risk.

**Fig 3:**
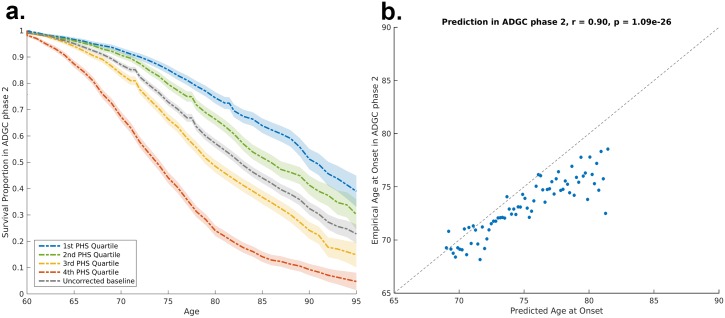
Polygenic hazard score validation in ADGC Phase 2 cohort. (A) Risk stratification in ADGC Phase 2 cohort, using PHSs derived from ADGC Phase 1 dataset. The dashed lines and shaded regions represent Kaplan–Meier estimations with 95% confidence intervals. (B) Predicted age of AD onset as a function of empirical age of AD onset among cases in ADGC Phase 2 cohort. Prediction is based on the final survival model trained in the ADGC Phase 1 dataset. AD, Alzheimer disease; ADGC, Alzheimer’s Disease Genetics Consortium; PHS, polygenic hazard score.

To assess replication, we applied the ADGC Phase 1–trained model to independent samples from ADGC Phase 2. Using the empirical distributions, we found that the PHS successfully stratified individuals from independent cohorts into different risk strata (Fig 3A). Among AD cases in the ADGC Phase 2 cohort, we found that the predicted age of onset was strongly associated with the empirical (actual) age of onset (binned in percentiles, *r* = 0.90, *p* = 1.1 × 10^−26^; Fig 3B). Similarly, within the NIA ADC subset with a high level of AD neuropathological change, we found that the PHS strongly predicted time to progression to neuropathologically defined AD (Cox proportional hazard model, *z* = 11.8723, *p* = 2.8 × 10^−32^).

### Predicting population risk of Alzheimer disease onset

To evaluate the risk for developing AD, combining the estimated hazard ratios from the ADGC cohort, allele frequencies for each of the AD-associated SNPs from the 1000 Genomes Project, and the disease incidence in the general US population [18], we generated population baseline-corrected survival curves given an individual’s genetic profile and age (panels A and B of second figure in S1 Appendix). We found that PHS status modifies both the risk for developing AD and the distribution of age of onset (panels A and B of second figure in S1 Appendix).

**Fig 4:**
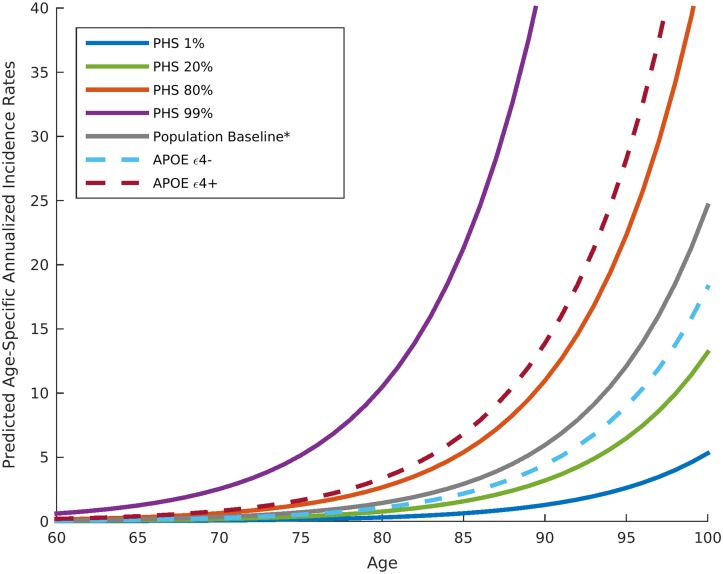
Annualized incidence rates showing the instantaneous hazard as a function of polygenic hazard score percentile and age. The gray line represents the population baseline estimate. Dashed lines represent incidence rates in *APOE* ε4 carriers (dark red dashed line) and non-carriers (light blue dashed line) not associated with a PHS percentile. The asterisk indicates that the baseline estimation is based on previously reported annualized incidence rates by age in the general US population [18]. PHS, polygenic hazard score.

**Fig 5:**
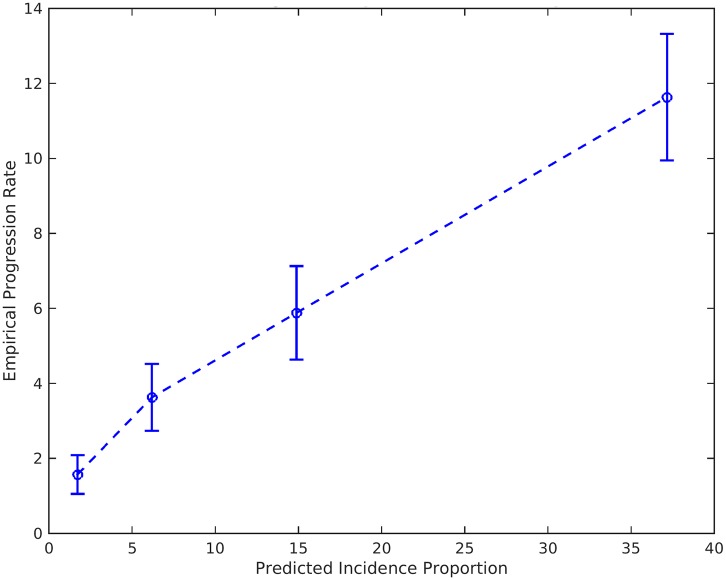
Empirical progression rates observed in the NIA ADC longitudinal cohort as a function of predicted incidence. Bars show 95% confidence intervals. NIA ADC, National Institute on Aging Alzheimer’s Disease Center.

Given an individual’s genetic profile and age, the corrected survival proportion can be translated directly into incidence rates (Fig 4; Tables 3 and S1). As previously reported in a meta-analysis summarizing four studies from the US general population [18], the annualized incidence rate represents the proportion (in percent) of individuals in a given risk stratum and age who have not yet developed AD but will develop AD in the following year; thus, the annualized incidence rate represents the instantaneous risk for developing AD conditional on having survived up to that point in time. For example, for a cognitively normal 65-y-old individual in the 80th percentile of the PHS, the incidence rate (per 100 person-years) would be 0.29 at age 65 y, 1.22 at age 75 y, 5.03 at age 85 y, and 20.82 at age 95 y (Fig 4; Table 3); in contrast, for a cognitively normal 65-y-old in the 20th percentile of the PHS, the incidence rate would be 0.10 at age 65 y, 0.43 at age 75 y, 1.80 at age 85 y, and 7.43 at age 95 y (Fig 4; Table 3). As independent validation, we examined whether the PHS-predicted incidence rate reflects the empirical progression rate (from normal control to clinical AD) (Fig 5). We found that the PHS-predicted incidence was strongly associated with empirical progression rates (Cochran–Armitage trend test, *p* = 1.5 × 10^−10^).

**Table 3:** Predicted annualized incidence rate (per 100 person-years) by age using polygenic hazard score.

Age (years)	Population baseline*	PHS 1st percentile (95% CI)	PHS 20th percentile (95% CI)	PHS 80th percentile (95% CI)	PHS 99th percentile (95% CI)	*APOE* ε4+ (95% CI)	*APOE* ε4− (95% CI)
60	0.08	0.02 (0.01, 0.03)	0.04 (0.01, 0.08)	0.15 (0.04, 0.27)	0.61 (0.16, 1.06)	0.19 (0.18, 0.20)	0.06 (0.06, 0.70)
65	0.17	0.04 (0.01, 0.06)	0.09 (0.03, 0.16)	0.32 (0.09, 0.54)	1.24 (0.33, 2.15)	0.38 (0.36, 0.40)	0.13 (0.12, 0.13)
70	0.35	0.07 (0.02, 0.13)	0.19 (0.05, 0.32)	0.64 (0.18, 1.10)	2.53 (0.68, 4.38)	0.78 (0.74, 0.82)	0.26 (0.25, 0.27)
75	0.71	0.15 (0.05, 0.19)	0.38 (0.11, 0.65)	1.30 (0.36, 2.25)	5.15 (1.38, 8.91)	1.58 (1.51, 1.66)	0.53 (0.52, 0.55)
80	1.44	0.31 (0.26, 0.26)	0.77 (0.22, 1.32)	2.65 (0.74, 4.57)	10.47 (2.81, 18.13)	3.22 (3.06, 3.38)	1.08 (1.05, 1.11)
85	2.92	0.63 (0.19, 1.07)	1.57 (0.45, 2.68)	5.39 (1.50, 9.29)	21.30 (5.72, 36.88)	6.55 (6.23, 6.87)	2.20 (2.13, 2.27)
90	5.95	1.28 (0.38, 2.18)	3.19 (0.91, 5.46)	10.97 (3.05, 18.89)	43.32 (11.63, 75.00)	13.33 (12.68, 13.98)	4.48 (4.34, 4.61)
95	12.10	2.61 (0.78, 4.44)	6.48 (1.85, 11.10)	22.31 (6.20, 38.43)	88.11 (23.66, 100.00)	27.11 (25.79, 28.43)	9.10 (8.83, 9.38)

*APOE* ε4+ refers to individuals with at least one copy of the ε4 allele of *APOE*; *APOE* ε4− refers to individuals with no copies of the ε4 allele of *APOE*.

*US community-sampled population incidence proportion (percent per year) reported by [18].

### Association of polygenic hazard score with known markers of Alzheimer disease pathology

We found that the PHS was significantly associated with Braak stage of NFTs (β-coefficient = 0.115, standard error [SE] = 0.024, *p-*value = 3.9 × 10^−6^) and CERAD score for neuritic plaques (β-coefficient = 0.105, SE = 0.023, *p-*value = 6.8 × 10^−6^). We additionally found that the PHS was associated with worsening CDR-SB score over time (β-coefficient = 2.49, SE = 0.38, *p-*value = 1.1 × 10^−10^), decreased CSF Aβ_1–42_ (reflecting increased intracranial Aβ plaque load) (β-coefficient = −0.07, SE = 0.01, *p-*value = 1.28 × 10^−7^), increased CSF total tau (β-coefficient = 0.03, SE = 0.01, *p-*value = 0.05), and greater volume loss within the entorhinal cortex (β-coefficient = −0.022, SE = 0.005, *p-*value = 6.30 × 10^−6^) and hippocampus (β-coefficient = −0.021, SE = 0.005, *p-*value = 7.86 × 10^−5^).

## Discussion

In this study, by integrating AD-associated SNPs from recent GWASs and disease incidence estimates from the US population into a genetic epidemiology framework, we have developed a novel PHS for quantifying individual differences in risk for developing AD, as a function of genotype and age. The PHS systematically modified age of AD onset, and was associated with known in vivo and pathological markers of AD neurodegeneration. In independent cohorts (including a neuropathologically confirmed dataset), the PHS successfully predicted empirical (actual) age of onset and longitudinal progression from normal aging to AD. Even among individuals who do not carry the ε4 allele of *APOE* (the majority of the US population), we found that polygenic information was useful for predicting age of AD onset.

Using a case–control design, prior work has combined GWAS-associated polymorphisms and disease prediction models to predict risk for AD [19–24]. Rather than representing a continuous process where non-demented individuals progress to AD over time, the case–control approach implicitly assumes that normal controls do not develop dementia and treats the disease process as a dichotomous variable where the goal is maximal discrimination between diseased “cases” and healthy “controls.” Given the striking age dependence of AD, this approach is clinically suboptimal for estimating the risk of AD. Building on prior genetic estimates from the general population [2,25], we employed a survival analysis framework to integrate AD-associated common variants with established population-based incidence [18] to derive a continuous measure, the PHS. We note that the PHS can estimate individual differences in AD risk across a lifetime and can quantify the yearly incidence rate for developing AD.

These findings indicate that the lifetime risk of age of AD onset varies by polygenic profile. For example, the annualized incidence rate (risk for developing AD in a given year) is considerably lower for an 80-y-old individual in the 20th percentile of the PHS than for an 80-y-old in the 99th percentile of the PHS (Fig 4; Table 3). Across the lifespan (panel B of second figure in S1 Appendix), our results indicate that even individuals with low genetic risk (low PHS) develop AD, but at a later peak age of onset. Certain loci (including *APOE* ε2) may “protect” against AD by delaying, rather than preventing, disease onset.

Our polygenic results provide important predictive information beyond *APOE*. Among *APOE* ε3/3 individuals, who constitute 70%–75% of all individuals diagnosed with late-onset AD, age of onset varies by more than 10 y, depending on polygenic risk profile (Fig 2). At 60% AD risk, *APOE* ε3/3 individuals in the first decile of the PHS have an expected age of onset of 85 y, whereas for individuals in the tenth decile of the PHS, the expected age of onset is greater than 95 y. These findings are directly relevant to the general population, where *APOE* ε4 accounts for only a fraction of AD risk [3], and are consistent with prior work [26] indicating that AD is a polygenic disease where non-*APOE* genetic variants contribute significantly to disease etiology.

We found that the PHS strongly predicted age of AD onset within the ADGC Phase 2 dataset and the NIA ADC neuropathology-confirmed subset, demonstrating independent replication of our polygenic score. Within the NIA ADC sample, the PHS robustly predicted longitudinal progression from normal aging to AD, illustrating that polygenic information can be used to identify the cognitively normal older individuals at highest risk for developing AD (preclinical AD). We found a strong relationship between the PHS and increased tau-associated NFTs and amyloid plaques, suggesting that elevated genetic risk may make individuals more susceptible to underlying AD pathology. Consistent with recent studies showing correlations between AD polygenic risk scores and markers of AD neurodegeneration [22,23], our PHS also demonstrated robust associations with CSF Aβ_1–42_ levels, longitudinal MRI measures of medial temporal lobe volume loss, and longitudinal CDR-SB scores, illustrating that increased genetic risk may increase the likelihood of clinical progression and developing neurodegeneration measured in vivo.

From a clinical perspective, our genetic risk score may serve as a “risk factor” for accurately identifying older individuals at greatest risk for developing AD, at a given age. Conceptually similar to other polygenic risk scores (for a review of this topic see [27]) for assessing coronary artery disease risk [28] and breast cancer risk [29], our PHS may help in predicting which individuals will test “positive” for clinical, CSF, or imaging markers of AD pathology. Importantly, a continuous polygenic measure of AD genetic risk may provide an enrichment strategy for prevention and therapeutic trials and could also be useful for predicting which individuals may respond to therapy. From a disease management perspective, by providing an accurate probabilistic assessment regarding the likelihood of AD neurodegeneration, determining a “genomic profile” of AD may help initiate a dialogue on future planning. Finally, a similar genetic epidemiology framework may be useful for quantifying the risk associated with numerous other common diseases.

There are several limitations to our study. We primarily focused on individuals of European descent. Given that AD incidence [30], genetic risk [25,31], and likely linkage disequilibrium in African-American and Latino individuals is different from in white individuals, additional work will be needed to develop a polygenic risk model in non-white (and non-US) populations. The majority of the participants evaluated in our study were recruited from specialized memory clinics or AD research centers and may not be representative of the general US population. In order to be clinically useful, we note that our PHS needs to be prospectively validated in large community-based cohorts, preferably consisting of individuals from a range of ethnicities. The previously reported population annualized incidence rates were not separately provided for males and females [18]. Therefore, we could not report PHS annualized incidence rates stratified by sex. We note that we primarily focused on genetic markers and thus did not evaluate how other variables, such as environmental or lifestyle factors, in combination with genetics impact age of AD onset. Another limitation is that our PHS may not be able to distinguish pure AD from a “mixed dementia” presentation since cerebral small vessel ischemic/hypertensive pathology often presents concomitantly with AD neurodegeneration, and additional work will be needed on cohorts with mixed dementia to determine the specificity of our polygenic score. Finally, we focused on *APOE* and GWAS-detected polymorphisms for disease prediction. Given the flexibility of our genetic epidemiology framework, it can be used to investigate whether a combination of common and rare genetic variants along with clinical, cognitive, and imaging biomarkers may prove useful for refining the prediction of age of AD onset.

In conclusion, by integrating population-based incidence proportion and genome-wide data into a genetic epidemiology framework, we have developed a PHS for quantifying the age-associated risk for developing AD. Measures of polygenic variation may prove useful for stratifying AD risk and as an enrichment strategy in clinical trials.

## Supporting information

S1 Acknowledgments Supplemental acknowledgments and funding information for IGAP, NIA ADS, ADGC, ADNI, and NACC. (DOCX)

S1 Appendix Supplemental methods and figures. (DOCX)

S1 Table Predicted annualized incidence rate (per 100 person-years) by age using polygenic hazard scores (full range of scores). (CSV)

S1 TRIPOD Checklist TRIPOD checklist. (DOCX)

## Abbreviations

AD: Alzheimer disease
ADGC: Alzheimer’s Disease Genetics Consortium
ADNI: Alzheimer’s Disease Neuroimaging Initiative
CDR-SB: Clinical Dementia Rating Sum of Boxes
CERAD: Consortium to Establish a Registry for Alzheimer’s Disease
CSF: cerebrospinal fluid
GWAS: genome-wide association studies
IGAP: International Genomics of Alzheimer’s Project
NACC: National Alzheimer’s Coordinating Center
NFTs: neurofibrillary tangles
NIA ADC: National Institute on Aging Alzheimer’s Disease Center
PHS: polygenic hazard score
SE: standard error
SNP: single nucleotide polymorphism
