# Correction: Genetic assessment of age-associated Alzheimer disease risk: Development and validation of a polygenic hazard score

**DOI:** 10.1371/journal.pmed.1002289

**Published:** 2017-03-28

**Authors:** The PLOS Medicine Staff

S1 Appendix is a duplicate of the manuscript text. Please view the correct S1 Appendix below. The publisher apologizes for the error.

## Supporting information

S1 Appendix Supplemental methods and figures. (DOCX)
