# Supplementary material for: Genetic assessment of age-associated Alzheimer disease risk: Development and validation of a polygenic hazard score
Source: PLoS Med. 2017 Mar 21;14(3):e1002258. doi: 10.1371/journal.pmed.1002258 (PMC5360219; doi:10.1371/journal.pmed.1002258)
Supplement: S1 Appendix — (DOCX) [file pmed.1002258.s002.docx]

Genetic assessment of age-associated Alzheimer’s disease risk: development and validation of a polygenic hazard score

Rahul S. Desikan, MD, PhD^1*#^; Chun Chieh Fan, MD^2*^; Yunpeng Wang, PhD^3,4^; Andrew J. Schork, MS^2^; Howard J. Cabral, PhD^5^; L. Adrienne Cupples, PhD^5^; Wesley K. Thompson, PhD^6^; Lilah Besser, MSPH^7^; Walter A. Kukull, PhD^7^; Dominic Holland, PhD^3^; Chi-Hua Chen, PhD^8^; James B. Brewer, MD, PhD^3,8,19^; David S. Karow, MD, PhD^8^; Karolina Kauppi, PhD^8^; Aree Witoelar, PhD^4^; Celeste M. Karch, PhD^9^; Luke W. Bonham, BS^10^; Jennifer S. Yokoyama, PhD^10^; Howard J. Rosen, MD^10^; Bruce L. Miller, MD^10^; William P. Dillon, MD^1^; David M. Wilson, MD, PhD^1^; Christopher P. Hess, MD, PhD^1^; Margaret Pericak-Vance, PhD^11^; Jonathan L. Haines, PhD^12^; Lindsay A. Farrer, PhD^13^; Richard Mayeux, MD^14^; John Hardy, PhD^15^; Alison M. Goate, PhD^16^; Bradley T. Hyman, MD, PhD^17^; Gerard D. Schellenberg, PhD^18^; Linda K. McEvoy, PhD^8^; Ole A. Andreassen, MD, PhD^4#^; Anders M. Dale, PhD^2,3,8#^

*Contributed equally

^1^Neuroradiology Section, Department of Radiology and Biomedical Imaging, University of California, San Francisco, San Francisco, CA USA

Departments of ^2^Cognitive Sciences and ^3^Neurosciences, University of California, San Diego, La Jolla, CA, USA

^4^NORMENT; Institute of Clinical Medicine, University of Oslo and Division of Mental Health and Addiction, Oslo University Hospital, Oslo, Norway

^5^Department of Biostatistics, Boston University School of Public Health, Boston, MA, USA

^6^Institute for Biological Psychiatry, Sankt Hans Psychiatric Hospital, Roskilde, Denmark

^7^National Alzheimer's Coordinating Center, Department of Epidemiology, University of Washington, Seattle, WA, USA

^8^Department of Radiology, University of California, San Diego, La Jolla, CA, USA

^9^Department of Psychiatry, Washington University, St. Louis, MO, USA

^10^Department of Neurology, University of California, San Francisco, San Francisco, CA, USA

^11^The John P. Hussman Institute for Human Genomics, University of Miami, Miami, Florida, USA

^12^Department of Epidemiology and Biostatistics and Institute for Computational Biology, Case Western University, Cleveland, Ohio, USA

^13^Departments of Medicine (Biomedical Genetics), Neurology, Ophthalmology, Biostatistics, and Epidemiology, Boston University Schools of Medicine and Public Health, Boston, Massachusetts, USA

^14^Department of Neurology, Taub Institute on Alzheimer's Disease and the Aging Brain, and Gertrude H. Sergievsky Center, Columbia University, New York, New York, USA

^15^Department of Molecular Neuroscience, UCL Institute of Neurology, London, UK

^16^Departments of Neuroscience, Genetics and Genomic Sciences, Icahn School of Medicine at Mount Sinai, New York, NY, USA

^17^Department of Neurology, Massachusetts General Hospital, Boston, MA, USA

^18^Department of Pathology and Laboratory Medicine, University of Pennsylvania Perelman School of Medicine, Philadelphia, PA, USA

^19^Shiley-Marcos Alzheimer’s Disease Research Center, University of California, San Diego, La Jolla, CA, USA

#Correspondence should be addressed to:

Dr. Rahul S. Desikan

Neuroradiology Section, L-352

University of California, San Francisco

505 Parnassus Avenue

San Francisco, CA, USA 94143

Email: rahul.desikan@ucsf.edu

Phone: (415)-353-1079

Dr. Ole A. Andreassen

KG Jebsen Centre for Psychosis Research

Building 49, Oslo University Hospital, Ullevål

Kirkeveien 166, PO Box 4956 Nydalen

0424 Oslo, Norway

Email: o.a.andreassen@medisin.uio.no

Ph: +47 23 02 73 50 (22 11 78 43 dir)

Fax: +47 23 02 73 33

Dr. Anders M. Dale

Department of Radiology

University of California, San Diego

8950 Villa La Jolla Drive, Suite C101

La Jolla, CA, USA 92037-0841

Emails: amdale@ucsd.edu

Phone: (858)-822-6671

Fax: (858)-534-1078

Manuscript = 3,360 words

**ABSTRACT**

**Background:** Identifying individuals at risk for developing Alzheimer’s disease (AD) is of utmost importance. Although genetic studies have identified *APOE* and other AD associated single nucleotide polymorphisms (SNPs), genetic information has not been integrated into an epidemiological framework for risk prediction.

**Methods and Findings:** Using genotype data from 17,008 AD cases and 37,154 controls from the International Genomics of Alzheimer’s Disease Project (IGAP Stage 1), we identified AD associated SNPs (at p < 10^-5^). We then integrated these AD associated SNPs into a Cox proportional hazards model using genotype data from a subset of 6,409 AD patients and 9,386 older controls from Phase 1 of the Alzheimer’s Disease Genetics Consortium (ADGC), providing a polygenic hazard score (PHS) for each participant. By combining population based incidence rates, and genotype-derived PHS for each individual, we derived estimates of instantaneous risk for developing AD, based on genotype and age, and tested replication in multiple independent cohorts (ADGC Phase 2, NIA-ADC, and ADNI total n = 20,680). Within the ADGC Phase 1 cohort, individuals in the highest PHS quantiles developed AD at a considerably lower age and had the highest yearly AD incidence rate. Among *APOE* ε3/3 individuals, PHS modified expected age of AD onset by more than 10 years between the lowest and highest deciles (HR: 3.34, 95% CI: 2.62 – 4.24, p = 1.0 x 10^-22^). In independent cohorts, PHS strongly predicted empirical age of AD onset (ADGC Phase 2, r = 0.90, p = 1.1 x 10^-26^), longitudinal progression from normal aging to AD (NIA-ADC, Cochrane Armitage trend test, p = 1.54 x 10^-10^), and associated with neuropathology (NIA-ADC, Braak stage of neurofibrillary tangles p-value = 3.9 x 10^-6^ and CERAD score for neuritic plaques p-value = 6.8 x 10^-6^), and *in vivo* markers of AD neurodegeneration (ADNI). Additional prospective validation of these results on non-US, non-Caucasian and prospective community-based cohorts is necessary.

**Conclusions:** We have developed a PHS for quantifying individual differences in age-specific genetic risk for AD. Within the cohorts studied here, polygenic architecture plays an important role in modifying AD risk beyond *APOE*. With thorough validation, quantification of inherited genetic variation may prove useful for stratifying AD risk and as an enrichment strategy in therapeutic trials.

**AUTHOR SUMMARY**

### Why Was This Study Done?

### Across the United States, late onset Alzheimer’s disease (AD) is the most common form of dementia.

### There is a strong need for *in vivo* markers for AD risk stratification and cohort enrichment in therapeutic trials.

### Although numerous studies have identified several genetic risk factors, including the ε4 allele of apolipoprotein E (*APOE*), genetic variants have not been integrated with genetic epidemiology for quantifying AD age of onset.

### What Did the Researchers Do and Find?

- Using genotype data from over 70,000 AD patients and normal elderly controls, we evaluated the feasibility of combining AD associated SNPs and *APOE* status into a continuous measure ‘polygenic hazard score’ for predicting the age-specific risk for developing AD.
- Using a survival model framework, we integrated SNPs associated with increased risk for AD into a polygenic hazard score (PHS) for each participant. By combining population based incidence rates, and genotype-derived PHS for each individual, we derived estimates of instantaneous risk for developing AD, based on genotype and age, and tested replication in two independent cohorts.
- Individuals in the highest PHS quantiles developed AD at a considerably lower age and had the highest yearly AD incidence rate.
- In independent cohorts, we found that PHS strongly predicted empirical age of AD onset, longitudinal progression from normal aging to AD, and associated strongly with neuropathology and *in vivo* markers of AD neurodegeneration.
- Additional prospective validation of these results on non-US, non-Caucasian and prospective community-based cohorts is necessary.

### What Do These Findings Mean?

- Genetic variants can be integrated within an epidemiology framework to derive a polygenic score that can quantify individual differences in age-specific genetic risk for AD, beyond *APOE*.
- Quantification of inherited genetic variation may prove useful for AD risk stratification and for therapeutic trials.

**INTRODUCTION**

Late onset Alzheimer’s disease (AD), the most common form of dementia, places a large emotional and economic burden on patients and society. With increasing health care expenditures among cognitively impaired elderly^1^, identifying individuals at risk for developing AD is of utmost importance for potential preventative and therapeutic strategies. Inheritance of the ε4 allele of apolipoprotein E (*APOE*) on chromosome 19q13 is the most significant risk factor for developing late-onset AD.^2^ *APOE* ε4 has a dose dependent effect on age of onset, increases AD risk three-fold in heterozygotes and fifteen-fold in homozygotes, and is implicated in 20-25% of patients with AD.^3^

In addition to *APOE*, recent genome-wide association studies (GWAS) have identified numerous AD associated single nucleotide polymorphisms (SNPs), most of which have a small effect on disease risk.^4-5^ Although no single polymorphism may be informative clinically, a combination of *APOE* and non-*APOE* SNPs may help identify older individuals at increased risk for AD. Despite the detection of novel AD associated genes, GWAS findings have not yet been incorporated into a genetic epidemiology framework for individualized risk prediction.

Building on a prior approach evaluating GWAS-detected genetic variants for disease prediction^7^ and using a survival analysis framework, we tested the feasibility of combining AD associated SNPs and *APOE* status into a continuous measure ‘polygenic hazard score’ (PHS) for predicting the age-specific risk for developing AD. We assessed replication of the PHS using several independent cohorts.

**METHODS**

*Participant Samples*

IGAP: To select AD associated SNPs, we evaluated publicly available AD GWAS summary statistic data (p-values and odds ratios) from the International Genomics of Alzheimer’s Disease Project (IGAP Stage 1, for additional details see Supporting Information and reference 4). For selecting AD associated SNPs, we used IGAP Stage 1 data, consisting of 17,008 AD cases and 37,154 controls drawn from 4 different consortia across North America and Europe (including the United States of America, England, France, Holland and Iceland) with genotyped or imputed data at 7,055,881 SNPs (for a description of the AD cases and controls within the IGAP Stage 1 sub-studies, please see Table 1 and reference 4).

ADGC: To develop the survival model for the polygenic hazard scores (PHS), we first evaluated age of onset and raw genotype data from 6,409 patients with clinically diagnosed AD and 9,386 cognitively normal older individuals provided by the Alzheimer’s Disease Genetics Consortium (ADGC, Phase 1, a subset of the IGAP dataset), excluding individuals from the National Institute of Aging Alzheimer’s Disease Center (NIA ADC) samples and Alzheimer’s Disease Neuroimaging Initiative (ADNI). To evaluate replication of PHS, we used an independent sample of 6,984 AD patients and 10,972 cognitively normal older individuals from the ADGC Phase 2 cohort (Table 1). A detailed description of the genotype and phenotype data within the ADGC datasets has been described in detail elsewhere.^7,24^ Briefly, the ADGC Phase 1 and 2 datasets (enrollment from 1984-2012) consist of case-control, prospective, and family-based sub-studies of Caucasian participants with AD occurrence after age 60 derived from the general community and Alzheimer’s disease centers across the US. Participants with autosomal dominant (*APP*, *PSEN1* and *PSEN2*) mutations were excluded. All participants were genotyped using commercially available high-density SNP microarrays from Illumina or Affymetrix. Clinical diagnosis of AD within the ADGC sub-studies was established using NINCDS/ADRDA criteria for definite, probable or possible AD. ^8^ For most participants, age of AD onset was obtained from medical records and defined as the age when AD symptoms manifested, as reported by the participant or an informant. For participants lacking age of onset, age at ascertainment was used. Patients with an age-at-onset or age-at-death less than 60 years, and Caucasians of European ancestry were excluded from the analyses. All ADGC Phase 1 and 2 control participants were defined within individual sub-studies as cognitively normal older adults at time of clinical assessment. The institutional review boards of all participating institutions approved the procedures for all ADGC sub-studies. Written informed consent was obtained from all participants or surrogates. For additional details regarding the ADGC datasets, please see references 7 and 24.

NIA ADC: To assess longitudinal prediction, we evaluated an ADGC-independent sample of 2,724 cognitively normal elderly individuals. Briefly, all participants were US based, evaluated at the National Institute of Aging (NIA) funded Alzheimer’s Disease Centers (ADCs) (data collection coordinated by the National Alzheimer’s Coordinating Center) and clinically followed for at least two years (enrollment = 1984 to 2012, evaluation years = 2005 to 2016). ^9^ Here, we focused on older individuals defined at baseline as having an overall Clinical Dementia Rating (CDR) of 0.0. To assess the relationship between polygenic risk and neuropathology, we assessed 2,960 participants from the NIA ADC samples with genotype and neuropathological evaluations. For the neuropathological variables, we examined the Braak stage for neurofibrillary tangles (NFTs) (0: none; I-II: entorhinal; III-IV: limbic, and V-VI: isocortical) ^10^ and the Consortium to Establish a Registry for Alzheimer’s Disease (CERAD) score for neuritic plaques (none/sparse, moderate, or frequent). ^11^ Finally, as an additional independent replication sample, we evaluated all NACC AD cases with genetic data who were classified at autopsy as having a ‘High’ level of AD neuropathologuc change (n = 361), based on the revised NIA-AA AD neuropathology criteria. ^25^ The institutional review boards of all participating institutions approved the procedures for all NIA ADC sub-studies. Written informed consent was obtained from all participants or surrogates.

ADNI: To assess the relationship between polygenic risk and *in vivo* biomarkers, we evaluated an ADGC-independent sample of 692 older controls, mild cognitive impairment and AD participants from the ADNI (see Supporting Information). Briefly, the ADNI is a multi-center, multisite, longitudinal study assessing clinical, imaging, genetic and biospecimen biomarkers from US-based participants through the process of normal aging to early mild cognitive impairment, to late mild cognitive impairment, to dementia or AD (see Supporting Information). Here, we focused specifically on participants from ADNI1 with cognitive, imaging and CSF assessments from 2003 to 2010. On a subset of ADNI1 participants with available genotype data, we evaluated baseline CSF levels of Aβ_1-42_ and total tau, as well as longitudinal clinical dementia rating-sum of box (CDR-SB) scores. In ADNI1 participants with available genotype and quality-assured baseline and follow-up MRI scans, we also assessed longitudinal sub-regional change in medial temporal lobe volume (atrophy) on 2471 serial T_1_-weighted MRI scans (for additional details see Supporting Information).

*Statistical Analysis*

We followed three steps to derive the polygenic hazard scores (PHS) for predicting AD age of onset: 1) we defined the set of associated SNPs, 2) we estimated hazard ratios for polygenic profiles, and 3) we calculated individualized absolute hazards (see Supporting Information for detailed description of these steps).

Using the IGAP Stage 1 sample, we first identified a list of SNPs associated with increased risk for AD, using a significance threshold of p < 10^-5^. Next, we evaluated all IGAP-detected, AD-associated SNPs within the ADGC Phase 1 case-control dataset. Using a stepwise procedure in survival analysis, we delineated the ‘final’ list of SNPs for constructing the polygenic hazard score. ^12-13^ Specifically, using Cox proportional hazard models, we identified the top AD-associated SNPs within the ADGC Phase 1 cohort (excluding NIA ADC and ADNI samples), while controlling for the effects of gender, *APOE* variants, and top five genetic principal components (to control for the effects of population stratification). We utilized age of AD onset and age of last clinical visit to estimate ‘age appropriate’ hazards ^14^ and derived a PHS for each participant. In each step of the stepwise procedure, the algorithm selected one SNP from the pool that most improved model prediction (i.e. minimizing the Martingale residuals); additional SNP inclusion that did not further minimize the residuals resulted in halting of the SNP selection process. To prevent over-fitting in this training step, we used 1000x bootstrapping for model averaging and estimating the hazard ratios for each selected SNPs. We assessed the proportional hazard assumption in the final model using graphical comparisons.

To assess for replication, we first examined whether the ADGC Phase 1 derived predicted PHSs could stratify individuals into different risk strata within the ADGC Phase 2 cohort. We next evaluated the relationship between predicted age of AD onset and the empirical/actual age of AD onset using cases from ADGC Phase 2. We binned risk strata into percentile bins and calculated the mean of actual age in that percentile as the empirical age of AD onset. In a similar fashion, we additionally tested replication within the NACC subset classified at autopsy as having a high level of AD neuropathologic change. ^25^

Because case-control samples cannot provide the proper baseline hazard, ^16^ we used the previously reported annualized incidence rates by age, estimated from the general United States of America (US) population. ^17^ For each participant, by combining the overall population-derived incidence rates ^17^ and genotype-derived PHS, we calculated an individual’s ‘instantaneous risk’ for developing AD, based on their genotype and age (for additional details see Supporting Information). To independently assess the predicted instantaneous risk, we evaluated longitudinal follow-up data from 2,724 cognitively normal older individuals from the NIA ADC with at least 2 years of clinical follow-up. We assessed the number of cognitively normal individuals progressing to AD as a function of the predicted PHS risk strata and examined whether the predicted PHS-derived incidence rate reflects the empirical/actual progression rate using a Cochran-Armitage trend test.

We examined the association between our PHS and established *in vivo* and pathologic markers of AD neurodegeneration. Using linear models, we assessed whether the PHS associated with Braak stage for NFTs and CERAD score for neuritic plaques as well as CSF Aβ_1-42_, and CSF total tau. Using linear mixed effects models, we also investigated whether the PHS was associated with longitudinal CDR-SB score and volume loss within the entorhinal cortex and hippocampus. In all analyses, we co-varied for the effects of age and sex.

**RESULTS**

*PHS: model development, relationship to APOE and independent replication*

From the IGAP cohort, we found 1854 SNPs associated with increased risk for AD at a p < 10^-5^. Of these, using the Cox stepwise regression framework, we identified 31 SNPs, in addition to two *APOE* variants, within the ADGC cohort for constructing the polygenic model (Table 2). Figure 1 illustrates the relative risk for developing AD using the ADGC case/control Phase 1 cohort. The graphical comparisons among Kaplan-Meier estimations and Cox proportional hazard models indicate the proportional hazard assumption holds for the final model (Figure 1).

To quantify the additional prediction provided by polygenic information beyond *APOE*, we evaluated how PHS modulates age of AD onset in *APOE* ε3/3 individuals. Among these individuals, we found that age of AD onset can vary by more than 10 years, depending on polygenic risk. For example, for an *APOE* ε3/3 individual in the 10^th^ decile (top 10%) of PHS, at 50% risk for meeting clinical criteria for AD diagnosis, the expected age for developing AD is approximately 84 years (Figure 2); however, for an *APOE* ε3/3 individual in the 1^st^ decile (bottom 10%) of PHS, the expected age of developing AD is approximately 95 years (Figure 2). The hazard ratio of 10^th^ decile to 1^st^ decile is 3.34 (95% CI: 2.62 - 4.24, logrank test: p = 1 x 10^-22^). Similarly, we also evaluated the relationship between PHS and the different *APOE* alleles (ε 2/3/4) (Supplemental Figure 1). These findings show that beyond *APOE*, the polygenic architecture plays an integral role in affecting AD risk.

To assess replication, we applied the ADGC Phase 1-trained model on independent samples from ADGC Phase 2. Using the empirical distributions, we found that the PHS successfully stratified individuals from independent cohorts into different risk strata (Figure 3a). Among AD cases in the ADGC Phase 2 cohort, we found that the predicted age of onset was strongly associated with the empirical (actual) age of onset (binned in percentiles, r = 0.90, p = 1.1 x 10^-26^, Figure 3b). Similarly within the NACC subset with a high level of AD neuropathologic change, we found that PHS strongly predicted time to progress to neuropathologically defined AD (Cox proportional hazard model, z = 11.8723, p = 2.82 x 10^-32^).

*Predicting population risk of AD onset*

To evaluate risk for developing AD, combining the estimated hazard ratios from the ADGC cohort, allele frequencies for each of the AD-associated SNPs from the 1000 Genomes Project and the disease incidence in the general US population, ^17^ we generated the population baseline-corrected survival curves given an individual’s genetic profile and age (Supplemental Figures 2A and 2B). We found that PHS status modifies both the risk for developing AD and the distribution of age of onset (Supplemental Figures 2A,B).

Given an individual’s genetic profile and age, the corrected survival proportion can be translated directly into incidence rates (Figure 4, Table 3 and Supplemental Table 1). As previously reported in a meta-analysis summarizing four studies from the US general population, ^17^ the annualized incidence rate represents the proportion (in percent) of individuals in a given risk stratum and age, who have not yet developed AD but will develop AD in the following year; thus the annualized incidence rate represents the instantaneous risk for developing AD conditional on having survived up to that point in time. For example, for a cognitively normal 65 year-old individual in the 80^th^ percentile PHS, the incidence rate would be: 0.29 at age 65, 1.22 at age 75, 5.03 at age 85, and 20.82 at age 95 (Figure 4 and Table 3); in contrast, for a cognitively normal 65 year old in the 20^th^ percentile PHS, the incidence rate (per 100 person-years) would be 0.10 at age 65, 0.43 at age 75, 1.80 at age 85, and 7.43 at age 95 (Figure 4 and Table 3). As independent validation, we examined whether the PHS predicted incidence rate reflects the empirical progression rate (from normal control to clinical AD) (Figure 5). We found that the PHS predicted incidence was strongly associated with empirical progression rates (Cochrane Armitage trend test, p = 1.54 x 10^-10^).

*Association with known markers of AD pathology*

We found that the PHS was significantly associated with Braak stage of NFTs (β-coefficient = 0.115, standard error (SE) = 0.024, p-value = 3.9 x 10^-6^) and CERAD score for neuritic plaques (β-coefficient = 0.105, SE = 0.023, p-value = 6.8 x 10^-6^). We additionally found that the PHS was associated with worsening CDR-Sum of Box score over time (β-coefficient = 2.49, SE = 0.38, p-value = 1.1 x 10^-10^), decreased CSF Aβ_1-42_ (reflecting increased intracranial Aβ plaque load) (β-coefficient = -0.07, SE = 0.01, p-value = 1.28 x 10^-7^), increased CSF total tau (β-coefficient = 0.03, SE = 0.01, p-value = 0.05), and increased volume loss within the entorhinal cortex (β-coefficient = -0.022, SE = 0.005, p-value = 6.30 x 10^-6^) and hippocampus (β-coefficient = -0.021, SE = 0.0054, p-value = 7.86 x 10^-5^).

**DISCUSSION**

In this study, by integrating AD-associated SNPs from recent GWAS and disease incidence estimates from the US population into a genetic epidemiology framework, we have developed a novel polygenic hazard score for quantifying individual differences in risk for developing AD, as a function of genotype and age. The PHS systematically modified age of AD onset, and was associated with known *in vivo* and pathologic markers of AD neurodegeneration. In independent cohorts (including a neuropathologically confirmed dataset), the PHS successfully predicted empirical (actual) age of onset and longitudinal progression from normal aging to AD. Even among individuals who do not carry the ε4 allele of *APOE* (the majority of the US population), we found that polygenic information is useful for predicting age of AD onset.

Using a case/control design, prior work has combined GWAS-associated polymorphisms and disease prediction models to predict risk for AD. ^18-19, 26-29^ Rather than representing a continuous process where non-demented individuals progress to AD over time, the case/control approach implicitly assumes that normal controls do not develop dementia and treats the disease process as a dichotomous variable where the goal is maximal discrimination between diseased ‘cases’ and healthy ‘controls’. Given the striking age-dependence of AD, this approach is clinically suboptimal for estimating risk of AD. Building on prior genetic estimates from the general population, ^2, 20^ we employed a survival analysis framework to integrate AD-associated common variants with established population-based incidence ^17^ to derive a continuous measure, polygenic hazard score (PHS). We note that the PHS can estimate individual differences in AD risk across a lifetime and can quantify the yearly incidence rate for developing AD.

These findings indicate that the lifetime risk of age of AD onset varies by polygenic profile. For example, the annualized incidence rates (risk for developing AD in a given year) are considerably lower for an 80-year old individual in the 20^th^ percentile PHS relative to an 80-year old in the 99^th^ percentile PHS (Figure 4 and Table 3). Across the lifespan (Supplemental Figure 2B), our results indicate that even individuals with low genetic risk (low PHS) develop AD, but at a later peak age of onset. Certain loci (including *APOE* ε2) may ‘protect’ against AD by delaying, rather than preventing, disease onset.

Our polygenic results provide important predictive information beyond *APOE*. Among *APOE* ε3/3 individuals, who constitute 70-75% of all individuals diagnosed with late-onset AD, age of onset varies by more than 10 years, depending on polygenic risk profile (Figure 2). At 60% AD risk *APOE* ε3/3 individuals in the 1^st^ decile of PHS have an expected age of onset of 85 whereas for individuals in the 10^th^ decile of PHS, the expected age of onset is greater than 95. These findings are directly relevant to the general population where *APOE* ε4 only accounts for a fraction of AD risk ^3^ and are consistent with prior work ^21^ indicating that AD is a polygenic disease where non-*APOE* genetic variants contribute significantly to disease etiology.

We found that the PHS strongly predicted age of AD onset within the ADGC phase 2 dataset and the NACC neuropathology confirmed subset demonstrating independent replication of our polygenic score. Within the NIA ADC sample, the PHS robustly predicted longitudinal progression from normal aging to AD illustrating that polygenic information can be used to identify cognitively normal older individuals at highest risk for developing AD (preclinical AD). We found a strong relationship between PHS and increased tau associated NFTs and amyloid plaques suggesting that elevated genetic risk may make individuals more susceptible to underlying Alzheimer’s pathology. Consistent with recent studies showing correlations between AD polygenic risk scores and markers of Alzheimer’s neurodegeneration, ^26-27^ our PHS also demonstrated robust associations with CSF Aβ_1-42_ levels, longitudinal MRI measures of medial temporal lobe volume loss and longitudinal CDR-SB scores illustrating that increased genetic risk may increase likelihood of clinical progression and developing neurodegeneration measured *in vivo*.

From a clinical perspective, our genetic risk score may serve as a ‘risk factor’ for accurately identifying older individuals at greatest risk for developing AD, at a given age. Conceptually similar to other polygenic risk scores (for a review of this topic see reference 30) for assessing coronary artery disease risk ^31^ or breast cancer, ^32^ our PHS may help in predicting which individuals may test ‘positive’ for clinical, CSF or imaging markers of AD pathology. Importantly, a continuous, polygenic measure of AD genetic risk may provide an enrichment strategy for prevention and therapeutic trials and could also be useful for predicting which individuals may respond to therapy. From a disease management perspective, by providing an accurate, probabilistic assessment regarding the likelihood of Alzheimer’s neurodegeneration, determining a ‘genomic profile’ of AD may help initiate a dialogue on future planning. Finally, a similar genetic epidemiology framework may be useful for quantifying the risk associated with numerous other common diseases.

There are several limitations to our study. We primarily focused on Caucasian individuals of European descent. Given that AD incidence ^20^, genetic risk ^22,23^ and likely linkage disequilibrium in African-Americans and Latinos is different from Caucasians, additional work will be needed to develop a polygenic risk model in non-Caucasian (and non-US) populations. The majority of the participants evaluated in our study were predominantly recruited from specialized memory clinics or AD research centers and may not be representative of the general US population. In order to be clinically useful, we note that our PHS needs to be prospectively validated in large community based cohorts, preferably consisting of individuals from a range of ethnicities. The previously reported population annualized incidence rates were not separately provided for males and females. ^17^ Therefore, we could not report PHS annualized incidence rates stratified by sex. We note that we primarily focused on genetic markers and thus did not evaluate how other variables, such as environmental or lifestyle factors, in combination with genetics, impact AD age of onset. Another limitation is that our PHS may not be able to distinguish pure AD from a ‘mixed dementia’ presentation since cerebral small vessel ischemic/hypertensive pathology often presents concomitantly with Alzheimer’s neurodegeneration and additional work will be needed on cohorts with mixed dementia to determine the specificity of our polygenic score. Finally, we focused on *APOE* and GWAS-detected polymorphisms for disease prediction. Given the flexibility of our genetic epidemiology framework, it can be used to investigate whether a combination of common and rare genetic variants along with clinical, cognitive and imaging biomarkers may prove useful for refining the prediction of AD age of onset.

In conclusion, by integrating population based incidence proportion and genome-wide data into a genetic epidemiology framework, we have developed a polygenic hazard score for quantifying the age-associated risk for developing AD. Measures of polygenic variation may prove useful for stratifying AD risk and as an enrichment strategy in clinical trials.

**ACKNOWLEDGEMENTS**

We thank the Shiley-Marcos Alzheimer’s Disease Research Center at UCSD, UCSF Memory and Aging Center and UCSF Center for Precision Neuroimaging for continued support, the International Genomics of Alzheimer's Project (IGAP) for providing summary results data for these analyses and the Alzheimer’s Disease Genetics Consortium (ADGC) and Alzheimer’s Disease Neuroimaging Initiative (ADNI) for providing data for these analyses. Please see Supplementary Acknowledgements for IGAP, NIAGADS, ADGC, ADNI and NACC funding sources.

**REFERENCES**

# Kelley AS, McGarry K, Gorges R, MA, Skinner JS. The Burden of Health Care Costs for Patients With Dementia in the Last 5 Years of Life. Ann Intern Med. 2015;163:729-736.

1. Farrer LA, Cupples LA, Haines JL, Hyman B, Kukull WA, Mayeux R, Myers RH, Pericak-Vance MA, Risch N, van Duijn CM. [Effects of age, sex, and ethnicity on the association between apolipoprotein E genotype and Alzheimer disease. A meta-analysis. APOE and Alzheimer Disease Meta Analysis Consortium.](http://www.ncbi.nlm.nih.gov/pubmed/9343467) JAMA. 1997 Oct 22-29;278(16):1349-56.

# Karch CM, Cruchaga C, Goate AM. [Alzheimer's disease genetics: from the bench to the clinic.](http://www.ncbi.nlm.nih.gov/pubmed/24991952) Neuron 2014;83:11-26.

1. Lambert JC, Ibrahim-Verbaas CA, Harold D et al. [Meta-analysis of 74,046 individuals identifies 11 new susceptibility loci for Alzheimer's disease.](http://www.ncbi.nlm.nih.gov/pubmed/24162737) Nat Genet 2013;45:1452-8.

# Desikan RS, Schork AJ, Wang Y, et al. [Polygenic Overlap Between C-Reactive Protein, Plasma Lipids, and Alzheimer Disease.](http://www.ncbi.nlm.nih.gov/pubmed/25862742) Circulation. 2015;131:2061-9.

1. Wray NR, Goddard ME, Visscher PM. [Prediction of individual genetic risk to disease from genome-wide association studies.](http://www.ncbi.nlm.nih.gov/pubmed/17785532) Genome Res 2007;17:1520-8.
2. Naj AC, Jun G, Beecham GW, Wang LS, et al. [Common variants at MS4A4/MS4A6E, CD2AP, CD33 and EPHA1 are associated with late-onset Alzheimer's disease.](http://www.ncbi.nlm.nih.gov/pubmed/21460841) Nat Genet 2011;43:436-41.
3. McKhann G, Drachman D, Folstein M, et al. [Clinical diagnosis of Alzheimer's disease: report of the NINCDS-ADRDA Work Group under the auspices of Department of Health and Human Services Task Force on Alzheimer's Disease.](http://www.ncbi.nlm.nih.gov/pubmed/6610841) Neurology 1984;34:939-44.

# Beekly DL, Ramos EM, Lee WW, et al. [The National Alzheimer's Coordinating Center (NACC) database: the Uniform Data Set.](http://www.ncbi.nlm.nih.gov/pubmed/17804958) Alzheimer Dis Assoc Disord 2007;21:249-58.

# Braak H, Braak E. [Neuropathological stageing of Alzheimer-related changes.](http://www.ncbi.nlm.nih.gov/pubmed/1759558) Acta Neuropathol 1991;82:239-59.

1. Mirra SS, Heyman A, McKeel D, Sumi SM, Crain BJ, Brownlee LM, Vogel FS, Hughes JP, van Belle G, Berg L. [The Consortium to Establish a Registry for Alzheimer's Disease (CERAD). Part II. Standardization of the neuropathologic assessment of Alzheimer's disease.](http://www.ncbi.nlm.nih.gov/pubmed/2011243) Neurology 1991;41:479-86.
2. Yang J, Ferreira T, Morris AP, et al. Conditional and joint multiple-SNP analysis of GWAS summary statistics identifies additional variants influencing complex traits. Nat Genet 2012;44:369-U170.
3. Dudbridge F. Power and Predictive Accuracy of Polygenic Risk Scores. Plos Genet 2013;9.

# Klein JP, Houwelingen HC, Ibrahim JG, Scheike TH. Handbook of Survival Analysis 2014.

1. Heagerty PJ, Zheng YY. Survival model predictive accuracy and ROC curves. Biometrics 2005;61:92-105.
2. Rothman KJ, Greenland S, Lash TL. Modern Epidemiology. 3rd ed: Lippincott Williams & Wilkins; 2008.

# Brookmeyer R, Gray S, Kawas C. [Projections of Alzheimer's disease in the United States and the public health impact of delaying disease onset.](http://www.ncbi.nlm.nih.gov/pubmed/9736873) Am J Public Health 1998;88:1337-42.

1. Escott-Price V, Sims R, Bannister C, et al. [Common polygenic variation enhances risk prediction for Alzheimer's disease.](http://www.ncbi.nlm.nih.gov/pubmed/26490334) Brain. 2015 Oct 21.
2. Yokoyama JS, Bonham LW, Sears RL, et al. [Decision tree analysis of genetic risk for clinically heterogeneous Alzheimer's disease.](http://www.ncbi.nlm.nih.gov/pubmed/25880661) BMC Neurol 2015;15:47.
3. Tang MX, Stern Y, Marder K, et al. [The APOE-epsilon4 allele and the risk of Alzheimer disease among African Americans, whites, and Hispanics.](http://www.ncbi.nlm.nih.gov/pubmed/9508150) JAMA 1998;279:751-5.
4. Sims R, Williams J. [Defining the Genetic Architecture of Alzheimer's Disease: Where Next.](http://www.ncbi.nlm.nih.gov/pubmed/26550988) Neurodegener Dis. 2016;16(1-2):6-11.
5. Tang MX, Cross P, Andrews H, Jacobs DM, et al. [Incidence of AD in African-Americans, Caribbean Hispanics, and Caucasians in northern Manhattan.](http://www.ncbi.nlm.nih.gov/pubmed/11148235) Neurology 2001;56:49-56.
6. Reitz C, Jun G, Naj A, et al. [Variants in the ATP-binding cassette transporter (ABCA7), apolipoprotein E ϵ4,and the risk of late-onset Alzheimer disease in African Americans.](http://www.ncbi.nlm.nih.gov/pubmed/23571587) JAMA 2013;309:1483-92.
7. Jun G, Ibrahim-Verbaas CA, Vronskaya M, et al. [A novel Alzheimer disease locus located near the gene encoding tau protein.](http://www.ncbi.nlm.nih.gov/pubmed/25778476) Mol Psychiatry. 2016 Jan;21:108-17.
8. Hyman BT, Phelps CH, Beach TG, et al. [National Institute on Aging-Alzheimer's Association guidelines for the neuropathologic assessment of Alzheimer's disease.](https://www-ncbi-nlm-nih-gov.ucsf.idm.oclc.org/pubmed/22265587) Alzheimers Dement. 2012 Jan;8(1):1-13.
9. Mormino EC, Sperling RA, Holmes AJ, et al. [Polygenic risk of Alzheimer disease is associated with early- and late-life processes.](https://www-ncbi-nlm-nih-gov.ucsf.idm.oclc.org/pubmed/27385740) Neurology. 2016 Aug 2;87(5):481-8.
10. Martiskainen H, Helisalmi S, Viswanathan J, et al. [Effects of Alzheimer's disease-associated risk loci on cerebrospinal fluid biomarkers and disease progression: a polygenic risk score approach.](https://www-ncbi-nlm-nih-gov.ucsf.idm.oclc.org/pubmed/25096612) J Alzheimers Dis. 2015;43(2):565-73.
11. Lacour A, Espinosa A, Louwersheimer E, et al. [Genome-wide significant risk factors for Alzheimer's disease: role in progression to dementia due to Alzheimer's disease among subjects with mild cognitive impairment.](https://www-ncbi-nlm-nih-gov.ucsf.idm.oclc.org/pubmed/26976043) Mol Psychiatry. 2016 Mar 15.
12. Chouraki V, Reitz C, Maury F, et al. [Evaluation of a Genetic Risk Score to Improve Risk Prediction for Alzheimer's Disease.](https://www-ncbi-nlm-nih-gov.ucsf.idm.oclc.org/pubmed/27340842) J Alzheimers Dis. 2016 Jun 18;53(3):921-32.
13. Chatterjee N, Shi J, García-Closas M. [Developing and evaluating polygenic risk prediction models for stratified disease prevention.](https://www-ncbi-nlm-nih-gov.ucsf.idm.oclc.org/pubmed/27140283) Nat Rev Genet. 2016 Jul;17(7):392-406.
14. Khera AV, Emdin CA, Drake I, et al. Genetic Risk, Adherence to a Healthy Lifestyle, and Coronary Disease. New England Journal of Medicine, November 13, 2016.
15. Mavaddat N, Pharoah PD, Michailidou K, et al. [Prediction of breast cancer risk based on profiling with common genetic variants.](https://www-ncbi-nlm-nih-gov.ucsf.idm.oclc.org/pubmed/25855707) J Natl Cancer Inst. 2015 Apr 8;107(5).

**Table 1**. Demographic data for AD patients and older controls.

|  | IGAP AD patients | IGAP older controls | ADGC Phase 1 AD patients | ADGC Phase 1 older controls | ADGC Phase  2 AD patients | ADGC Phase  2 older controls |
| --- | --- | --- | --- | --- | --- | --- |
| Total N | 17,008 | 37,154 | 6,409 | 9,386 | 6,984 | 10,972 |
| Mean age (SD) of onset (cases) or assessment (controls) | 74.7 (8.0) | 68.6 (8.5) | 74.7 (7.7) | 76.4 (8.1) | 73.6  (7.3) | 75.7  (8.6) |
| % Female | 63 | 57 | 61 | 59 | 57.6 | 60.7 |
| *% APOE* ε4 carriers | 59.0 | 25.4 | 51.6 | 26.7 | 56.0 | 28.4 |

**Table 2.** Selected 31 SNPs, their closest genes, log hazard ratio estimates, and their conditional p values in the final joint model, after controlling for effects of gender and APOE variants.

|  | **Chr** | **Position** | **Gene** | **β log HR ** | **Conditional p in –log_10_** |
| --- | --- | --- | --- | --- | --- |
| ε2 allele | 19 |  | *APOE* | -0.47 | > 15 |
| ε4 allele | 19 |  | *APOE* | 1.03 | > 20 |
| rs4266886 | 1 | 207685786 | *CR1* | -0.09 | 2.7 |
| rs61822977 | 1 | 207796065 | *CR1* | -0.08 | 2.8 |
| rs6733839 | 2 | 127892810 | *BIN1* | -0.15 | 10.5 |
| rs10202748 | 2 | 234003117 | *INPP5D* | -0.06 | 2.1 |
| rs115124923 | 6 | 32510482 | *HLA-DRB5* | 0.17 | 7.4 |
| rs115675626 | 6 | 32669833 | *HLA-DQB1* | -0.11 | 3.2 |
| rs1109581 | 6 | 47678182 | *GPR115* | -0.07 | 2.6 |
| rs17265593 | 7 | 37619922 | *BC043356* | -0.23 | 3.6 |
| rs2597283 | 7 | 37690507 | *BC043356* | 0.28 | 4.7 |
| rs1476679 | 7 | 100004446 | *ZCWPW1* | 0.11 | 4.9 |
| rs78571833 | 7 | 143122924 | *AL833583* | 0.14 | 3.8 |
| rs12679874 | 8 | 27230819 | *PTK2B* | -0.09 | 4.2 |
| rs2741342 | 8 | 27330096 | *CHRNA2* | 0.09 | 2.9 |
| rs7831810 | 8 | 27430506 | *CLU* | 0.09 | 3.0 |
| rs1532277 | 8 | 27466181 | *CLU* | 0.21 | 8.3 |
| rs9331888 | 8 | 27468862 | *CLU* | 0.16 | 5.1 |
| rs7920721 | 10 | 11720308 | *CR595071* | -0.07 | 2.9 |
| rs3740688 | 11 | 47380340 | *SPI1* | 0.07 | 2.8 |
| rs7116190 | 11 | 59964992 | *MS4A6A* | 0.08 | 3.9 |
| rs526904 | 11 | 85811364 | *PICALM* | -0.20 | 2.3 |
| rs543293 | 11 | 85820077 | *PICALM* | 0.30 | 4.2 |
| rs11218343 | 11 | 121435587 | *SORL1* | 0.18 | 2.8 |
| rs6572869 | 14 | 53353454 | *FERMT2* | -0.11 | 3.0 |
| rs12590273 | 14 | 92934120 | *SLC24A4* | 0.10 | 3.5 |
| rs7145100 | 14 | 107160690 | *abParts* | 0.08 | 2.0 |
| rs74615166 | 15 | 64725490 | *TRIP4* | -0.23 | 3.1 |
| rs2526378 | 17 | 56404349 | *BZRAP1* | 0.09 | 4.9 |
| rs117481827 | 19 | 1021627 | *C19orf6* | -0.09 | 2.5 |
| rs7408475 | 19 | 1050130 | *ABCA7* | 0.18 | 4.3 |
| rs3752246 | 19 | 1056492 | *ABCA7* | -0.25 | 8.4 |
| rs7274581 | 20 | 55018260 | *CASS4* | 0.10 | 2.1 |

**Table 3. Predicted annualized incidence rate (per 100 person-years) by age using polygenic hazard scores.**

| **Age** | **Population Baseline*** | **PHS 1 percentile (95% CI)** | **PHS 20^th^ percentile (95% CI)** | **PHS 80^th^ percentile (95% CI)** | **PHS 99^th^ percentile (95% CI)** | ***APOE* ε4+ (95% CI)** | ***APOE* ε4- (95% CI)** |
| --- | --- | --- | --- | --- | --- | --- | --- |
| 60 | 0.08 | 0.02  (0.01,0.03) | 0.04  (0.01,0.08) | 0.15  (0.04, 0.27) | 0.61  (0.16, 1.06) | 0.19  (0.18, 0.20) | 0.06  (0.06, 0.7) |
| 65 | 0.17 | 0.04  (0.01,0.06) | 0.09  (0.03, 0.16) | 0.32  (0.09, 0.54) | 1.24  (0.33,2.15) | 0.38  (0.36, 0.40) | 0.13  (0.12, 0.13) |
| 70 | 0.35 | 0.07  (0.02,0.13) | 0.19  (0.05,0.32) | 0.64  (0.18, 1.10) | 2.53  (0.68, 4.38) | 0.78  (0.74, 0.82) | 0.26  (0.25, 0.27) |
| 75 | 0.71 | 0.15  (0.05,0.19) | 0.38  (0.11,0.65) | 1.30  (0.36,2.25) | 5.15  (1.38, 8.91) | 1.58  (1.51, 1.66) | 0.53  (0.52, 0.55) |
| 80 | 1.44 | 0.31  (0.26,0.26) | 0.77  (0.22,1.32) | 2.65  (0.74, 4.57) | 10.47  (2.81, 18.13) | 3.22  (3.06, 3.38) | 1.08  (1.05, 1.11) |
| 85 | 2.92 | 0.63  (0.19,1.07) | 1.57  (0.45, 2.68) | 5.39  (1.50, 9.29) | 21.30  (5.72, 36.88) | 6.55  (6.23, 6.87) | 2.2  (2.13, 2.27) |
| 90 | 5.95 | 1.28  (0.38,2.18) | 3.19  (0.91, 5.46) | 10.97  (3.05, 18.89) | 43.32  (11.63, 75.00) | 13.33  (12.68, 13.98) | 4.48  (4.34, 4.61) |
| 95 | 12.1 | 2.61  (0.78,4.44) | 6.48  (1.85, 11.10) | 22.31  (6.20, 38.43) | 88.11  (23.66, 100.00) | 27.11  (25.79, 28.43) | 9.1  (8.83, 9.38) |

* US community-sampled population incidence proportion (% year) reported by reference 17.

# *APOE* ε4**+** refers to individuals with at least one copy of the ε4 allele of *APOE*; *APOE* ε4**-** refers to individuals with no copies of the ε4 allele of *APOE*

**FIGURE LEGENDS**

**Figure 1.** Kaplan-Meier estimates and Cox proportional model fits from the case-control ADGC phase 1 dataset, excluding NACC and ADNI samples. The proportional hazard assumptions were checked based on the graphical comparisons between Kaplan-Meier estimation (dashed line) and Cox proportional hazard models (solid line). 95% confidence intervals of Kaplan-Meier estimation are also demonstrated (shaded with corresponding colors). The baseline hazard (gray line) in this model is based on the mean of ADGC data.

**Figure 2.** Kaplan-Meier estimates and Cox proportional model fits among *APOE* ε3/ε3 individuals in ADGC phase 1 dataset, excluding NACC and ADNI samples. The solid line represent the Cox fit whereas the dashed line and shaded regions represent Kaplan-Meier estimation with 95% confidence interval.

**Figure 3.** **(a)** Risk stratification in ADGC phase 2 cohort, using PHS derived from ADGC phase 1 dataset. **(b)** Predicted age of AD onset as a function of empirical age of AD onset among cases in ADGC phase 2 cohort. Prediction is based on the final survival model trained in the ADGC phase 1 dataset. The dashed line and shaded regions represent Kaplan-Meier estimation with 95% confidence interval.

**Figure 4.** Annualized incidence rates showing the instantaneous hazard as a function of PHS percentiles and age. The gray line represents the population baseline estimate. Dashed lines represent incidence rates in *APOE* ε4 carriers (blue dashed line) and non-carriers (dark red dashed line) not associated with a PHS percentile.

**Figure 5.** Empirical progression rates observed in the NIA ADC longitudinal cohort as a function of predicted incidence. CA = Cochrane-Armitage test
