# Supplementary material for: Correction: Genetic assessment of age-associated Alzheimer disease risk: Development and validation of a polygenic hazard score
Source: PLoS Med. 2017 Mar 28;14(3):e1002289. doi: 10.1371/journal.pmed.1002289 (PMC5370097; doi:10.1371/journal.pmed.1002289)

**SUPPLEMENTAL INFORMATION**

**SUPPLEMENTAL METHODS**

***IGAP Cohort***

International Genomics of Alzheimer's Project (IGAP)^1^ is a large two-stage study based upon genome-wide association studies (GWAS) on individuals of European ancestry. In stage 1, IGAP used genotyped and imputed data on 7,055,881 single nucleotide polymorphisms (SNPs) to meta-analyse four previously-published GWAS datasets consisting of 17,008 Alzheimer's disease cases and 37,154 controls (The European Alzheimer's disease Initiative – EADI the Alzheimer Disease Genetics Consortium – ADGC The Cohorts for Heart and Aging Research in Genomic Epidemiology consortium – CHARGE The Genetic and Environmental Risk in AD consortium – GERAD). In stage 2, 11,632 SNPs were genotyped and tested for association in an independent set of 8,572 Alzheimer's disease cases and 11,312 controls. Finally, a meta-analysis was performed combining results from stages 1 & 2.

The institutional review boards of all participating institutions approved the procedures for IGAP substudies. Written informed consent was obtained from all participants or surrogates.

***ADNI cohort***

Data used in the preparation of this article were obtained from the Alzheimer’s Disease Neuroimaging Initiative (ADNI) database (adni.loni.usc.edu). The ADNI was launched in 2003 as a public-private partnership, led by Principal Investigator Michael W. Weiner, MD. The primary goal of ADNI has been to test whether serial magnetic resonance imaging (MRI), positron emission tomography (PET), other biological markers, and clinical and neuropsychological assessment can be combined to measure the progression of mild cognitive impairment (MCI) and early Alzheimer’s disease (AD).

Each participant was formally evaluated using eligibility criteria that are described in detail elsewhere (<http://www.adni-info.org/index.php?option=com_content&task=view&id=9&Itemid=43>). The institutional review boards of all participating institutions approved the procedures for this study. Written informed consent was obtained from all participants or surrogates. Experienced clinicians conducted independent semi-structured interviews with the participant and a knowledgeable collateral source that included a health history, neurological examination, and a comprehensive neuropsychological battery. We selected participants from the ADNI database if they were clinically diagnosed at baseline as cognitively normal, amnestic mild cognitive impairment (MCI) as defined using the revised MCI criteria or probable AD.

***Deriving Polygenic Hazard Scores***

Selecting set of previously implicated SNPs

We utilized previously published summary statistics (p-values and odds ratios) for AD associated SNPs from the IGAP consortium.^1^ Because the effect size estimates from the GWAS represent odds ratios that do not contain the time-to-event information, we did not use the estimated effect size from IGAP consortium. Instead, we only used the SNP list as a triage to filtering out most of unrelated SNPs. We set the significance threshold to p-value < 10^-5^ as the criteria for association with AD status. Among all IGAP stage 1 SNPs, we found 1854 SNPs with p-values < 10^-5^. From this list, we then used a forward stepwise regression to select SNPs for the final Cox proportional model. The final SNP selection process was conducted using the ADGC phase 1 cohort, excluding samples from NACC and ADNI. We further imputed with the missing genotypes in cohorts as the population mean, treating them as missing at random.

Forward stepwise Cox regression with conditional partial likelihood

To build the predictive model for AD age of onset, we adopted an approach that reduced the effect of linkage disequilibrium and further examined the effect of genetic loci on survival time. In each step, the algorithm includes one SNP that most minimized the Martingale regression residuals, and halts if no SNPs can further minimize the residuals. The Cox regressions were fitted with conditional partial likelihood:

$$L\left( \beta\right)= \prod_{j=1}^{d} \frac{exp(\beta^{'}x_{i})}{\sum_{k\in\tilde{R}{(t}_{j})} exp(\beta^{'}x_{k})}$$

Here, d represents each distinct time-event and R is the sampled risk set in the event time t_j_. Time-ties were solved with Efron’s method. Throughout the fitting process, we controlled for the effects of gender, *APOE* variants, and the top five genetic principal components. The selection procedures were conducted on the ADGC phase 1 cohort. In the final model, we identified 31 SNPs, in addition to the two APOE variants, that were associated with AD age of onset.

Calculating individualized absolute hazards

Instead of estimating the baseline hazard from the ADGC cohort, which is biased due to over-sampling of cases, we used the previously reported annualized incidence rates by age^2^ and then converted this into disease onset rates, R, as:

$$R(age)=1-S(age)\approx1-exp(-\sum_{k=60}^{age} I_{k})$$

S is the survival function, k is the age strata from 60 years old to *age* in years and the I_k_ is the annualized incidence rates given the age strata k. Combining this baseline hazard with the hazard ratios estimates from the previous section, we then derived the corrected survival function that predicts an individual’s risk of developing AD, given their polygenic profile and age. The incidence rates were also predicted given the individuals’ genetic background and their population baseline, as the annualized rate is the instantaneous hazard function converted from the corrected survival function:

$$Annualized Incidence rates= \lambda_{0}exp(X\beta)$$

For λ_0_ is the instantaneous hazard function from previous reports^2^, the $\beta$ is the Cox coefficients estimated in previous steps, assuming the genotypes X were centered on population mean. We used allele frequencies from 503 European descendants from the 1000 Genome Project to ensure proper centering and correct for sampling bias. We estimated the uncertainty of predicted incidence rates solely on the model estimates from ADGC cohorts because the previous published US population baseline annualized incidence rate estimates^2^ did not provide confidence intervals. Assuming $\hat{X\beta}$is asymptotically normal, centering on 0, the variance of predicted incidence proportions were derived using the delta method.

***Assessing Prediction Accuracy in Independent Cohorts***

We used one independent case-control sample, ADGC phase 2, and one longitudinal cohort, NACC, to evaluate the performance of PHS. First we used the PHS to generate the *predicted* age at onset in ADGC phase 2, and compared that to the corresponding *empirical* age at onset observed in ADGC phase 2. The observations were binned into 100 percentile bins to ensure the reliability of the empirical age at onset. Pearson correlations were calculated for the linear relationships between predicted and empirical age at onset in the ADGC phase 2. To take the censoring process into consideration, we also examined whether the PHS provides information for differentiating risk strata in the survival context. We used the likelihood ratio test to determine whether the PHS predicted the proportional risk in ADGC phase 2, while visualizing the stratification using Kaplan-Meier curves given strata in quartiles.

We then used the NACC longitudinal cohort to determine whether the PHS predicted progression from normal aging to AD. We focused on cognitively normal individuals who were followed for at least two years. Given the risk strata defined by PHS, we assessed whether the *rate* of AD onset is proportional to the increment of PHS. We used the Cochrane-Armitage test for trends to evaluate the association between PHS and empirical progression rate of AD.

***MR Image Processing***

All ADNI MRI scans were acquired at multiple sites using either a GE, Siemens, or Philips 1.5T system. Parameter values vary depending on scanning site and can be found http://adni.loni.usc.edu/. Multiple high-resolution T1- weighted volumetric MRI scans were collected for each subject and the raw DICOM images were downloaded from the public ADNI site (<http://adni.loni.usc.edu/data-samples/access-data/>). All MRI scans were analyzed using a modified version of the FreeSurfer software package (<http://surfer.nmr.mgh.harvard.edu>). These analysis procedures have been applied, validated, and described in detail in a number of publications. ^3^ In brief, the MRI scans were reviewed for quality, automatically corrected for spatial distortion due to gradient nonlinearity ^4^, registered and averaged to improve the signal to noise ratio. The cortical surface was automatically reconstructed ^5,6^ and gray matter thickness measurements were obtained at each point across the cortical mantle. ^7^

In this study, we primarily focused on the entorhinal cortex and hippocampus because AD-specific pathology is evident in this region in the earliest stages of the disease process. ^8-10^ The entorhinal cortex was delineated using an automated, surface-based parcellation atlas. ^11^ The hippocampus was identified using an automated, subcortical, segmentation atlas. ^12^ For the analysis of the longitudinal volume change, gray matter thickness change was examined using Quarc (quantitative anatomical regional change), a recently developed method from our laboratory. ^13-14^ Briefly, each participant's follow-up image was affine-aligned to the baseline scan and locally intensity-normalized. Using nonlinear registration, a deformation field was then calculated to locally register the images with high fidelity for both large- and small-scale structures, including those with low boundary contrast. From the deformation field, a volume-change field (atrophy) can directly be calculated. Using the baseline subcortical and cortical labels, the volume-change field can be sampled at points across the cortical surface or averaged over subcortical regions to give the percent volume change for those regions of interest.

**REFERENCES**

1. Lambert JC, Ibrahim-Verbaas CA, Harold D et al. [Meta-analysis of 74,046 individuals identifies 11 new susceptibility loci for Alzheimer's disease.](http://www.ncbi.nlm.nih.gov/pubmed/24162737) Nat Genet 2013;45:1452-8.
2. Brookmeyer R, Gray S, Kawas C. [Projections of Alzheimer's disease in the United States and the public health impact of delaying disease onset.](http://www.ncbi.nlm.nih.gov/pubmed/9736873) Am J Public Health 1998;88:1337-42.
3. Fennema-Notestine C, Hagler DJ Jr, McEvoy LK, Fleisher AS, Wu EH, Karow DS, et al. [Structural MRI biomarkers for preclinical and mild Alzheimer's disease.](http://www.ncbi.nlm.nih.gov.ezp-prod1.hul.harvard.edu/pubmed/19277975) Hum Brain Mapp. 2009;30:3238-53.
4. Jovicich J, Czanner S, Greve D, Haley E, van der Kouwe A, Gollub R, et al. [Reliability in multi-site structural MRI studies: effects of gradient non-linearity correction on phantom and human data.](http://www.ncbi.nlm.nih.gov/pubmed/16300968) Neuroimage. 2006;30:436-43.
5. Dale AM, Fischl B, Sereno MI. [Cortical surface-based analysis. I. Segmentation and surface reconstruction.](http://www.ncbi.nlm.nih.gov/pubmed/9931268) Neuroimage. 1999;9:179-94.
6. Fischl B, Sereno MI, Dale AM. [Cortical surface-based analysis. II: Inflation, flattening, and a surface-based coordinate system.](http://www.ncbi.nlm.nih.gov.ezp-prod1.hul.harvard.edu/pubmed/9931269) Neuroimage. 1999;9:195-207.
7. Fischl B, Dale AM. [Measuring the thickness of the human cerebral cortex from magnetic resonance images.](http://www.ncbi.nlm.nih.gov/pubmed/10984517) Proc Natl Acad Sci. 2000;97:11050-5.
8. Arriagada PV, Growdon JH, Hedley-Whyte ET, Hyman BT. [Neurofibrillary tangles but not senile plaques parallel duration and severity of Alzheimer's disease.](http://www.ncbi.nlm.nih.gov.ezp-prod1.hul.harvard.edu/pubmed/1549228) Neurology. 1992;42:631-9.
9. Braak H, Braak E. [Neuropathological stageing of Alzheimer-related changes.](http://www.ncbi.nlm.nih.gov.ezp-prod1.hul.harvard.edu/pubmed/1759558) Acta Neuropathol. 1991;82:239-59.
10. Gómez-Isla T, Price JL, McKeel DW Jr, Morris JC, Growdon JH, Hyman BT. [Profound loss of layer II entorhinal cortex neurons occurs in very mild Alzheimer's disease.](http://www.ncbi.nlm.nih.gov.ezp-prod1.hul.harvard.edu/pubmed/8699259) J Neurosci. 1996;16:4491-500.
11. Desikan RS, Ségonne F, Fischl B, Quinn BT, Dickerson BC, Blacker D, et al. An automated labeling system for subdividing the human cerebral cortex on MRI scans into gyral based regions of interest. Neuroimage, 2006;31:968-80.
12. Fischl B, Salat DH, Busa E, Albert M, Dieterich M, Haselgrove C, et al. [Whole brain segmentation: automated labeling of neuroanatomical structures in the human brain.](http://www.ncbi.nlm.nih.gov.ezp-prod1.hul.harvard.edu/pubmed/11832223) Neuron. 2002;33:341-55.
13. Holland D, Brewer JB, Hagler DJ, Fenema-Notestine C, Dale AM. [Subregional neuroanatomical change as a biomarker for Alzheimer's disease.](http://www.ncbi.nlm.nih.gov.ezp-prod1.hul.harvard.edu/pubmed/19996185) Proc Natl Acad Sci. 2009;106:20954-20959.
14. Holland D and Dale AM. Nonlinear registration of longitudinal images and measurement of change in regions of interest. Medical Image Analysis, 2011;15.4:489-97.

**SUPPLEMENTAL FIGURE**

**Supplemental Figure 1.** Frequency distribution of *APOE* ε 2/3/4 alleles as a function of PHS and years to AD onset (Age). As illustrated, the risk of developing AD corresponds to increasing PHS. Positive age values represent years free of AD and negative age values represent age to AD onset.

**Supplemental Figure 2**. **(A)** Survival functions stratified by polygenic hazard scores (PHS) and *APOE* ε4 status. The survival functions were predicted based on the population baseline and the estimated hazard ratios from the Cox regression. The survival functions are stratified according to the PHS percentiles and *APOE* ε4 carrier status, where *APOE* ε4+ represents ‘carriers’ and *APOE* ε4- represents ‘non-carriers’. **(B)** Predicted AD age of onset distribution. Based on the population baseline corrected survival function, the distribution of AD age of onset is illustrated.

**Supplemental Figure 1**

**
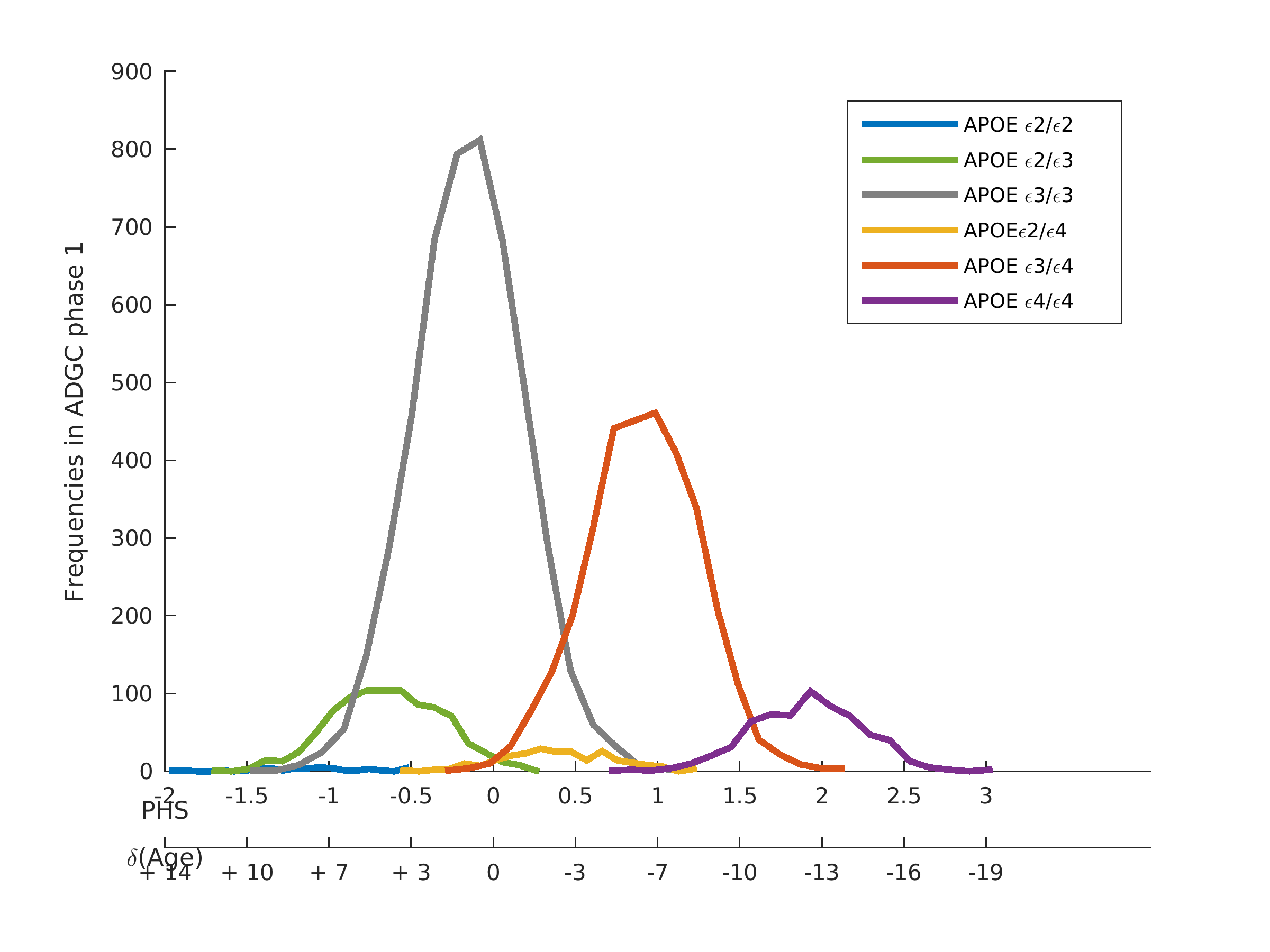
**

**Supplemental Figure 2A.**


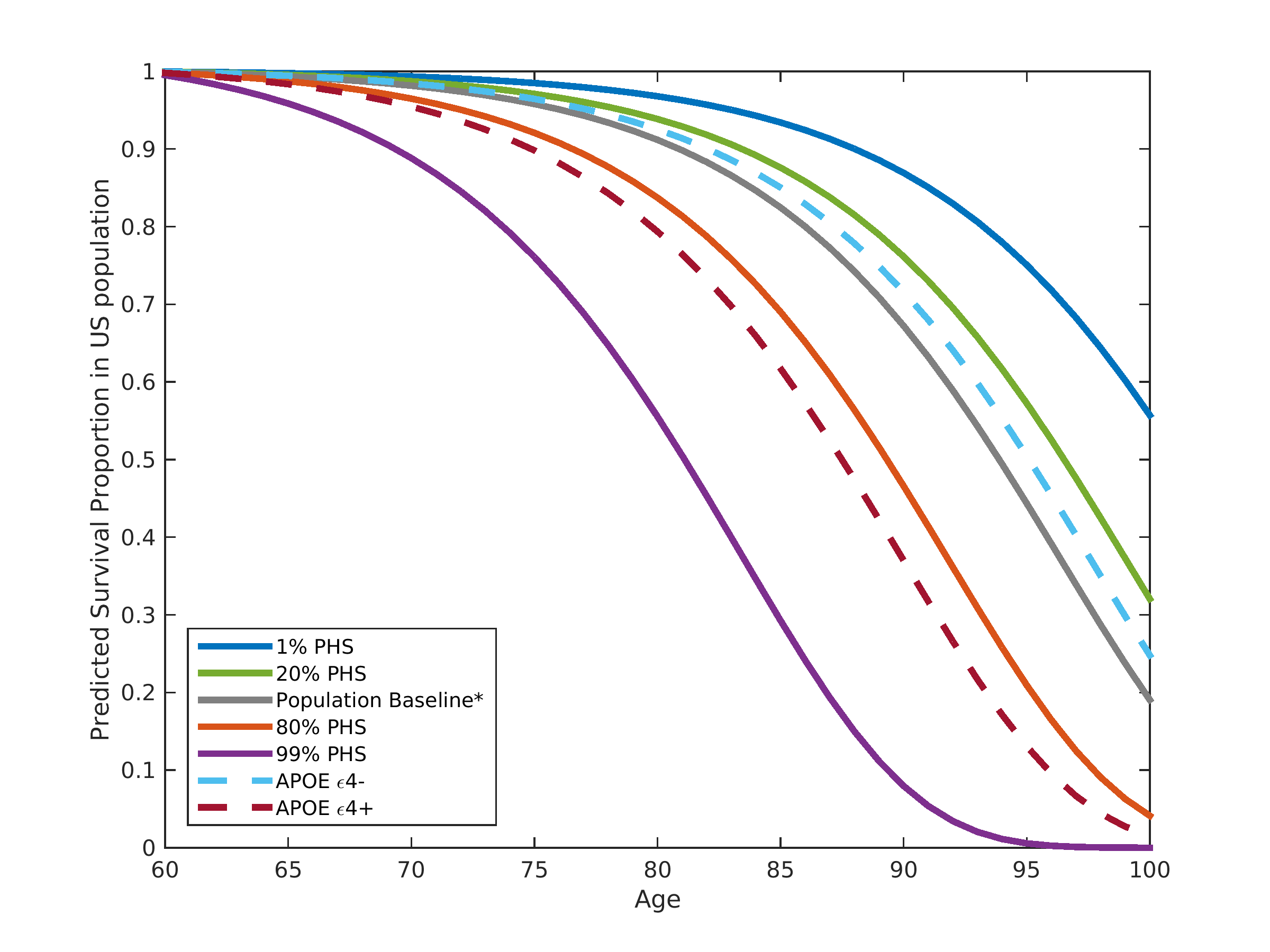


**Supplemental Figure 2B.**


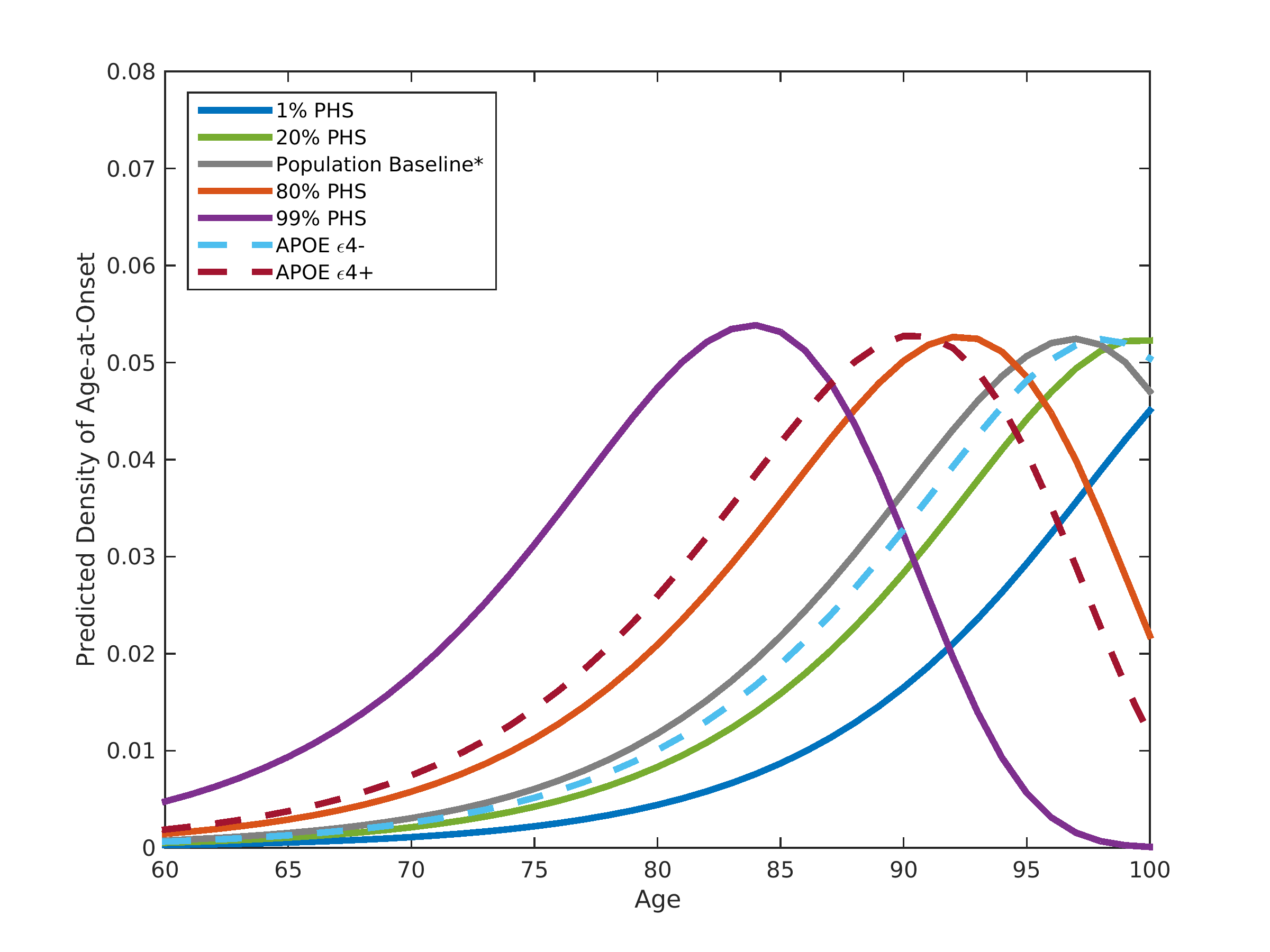

Supplement: S1 Appendix — (DOCX) [file pmed.1002289.s001.docx]
